# Accessory genome dynamics shape lineage-associated diversification in plum-associated *Xylella fastidiosa* subsp. *multiplex*

**DOI:** 10.1093/ismeco/ycag291

**Published:** 2026-10-13

**Authors:** Olakunle I Olawole, Cuong V Hoang, Ryan J Quaal, Alice F Olawole, Hyeonheui Ham, Lindsey P Burbank, Jianchi Chen, Brandon H Le, Pedro Uribe, Kevin Ong, Carlos F Gonzalez

**Affiliations:** Department of Microbiology & Plant Pathology, University of California, Riverside, CA 92521, United States; Department of Microbiology & Plant Pathology, University of California, Riverside, CA 92521, United States; Department of Microbiology & Plant Pathology, University of California, Riverside, CA 92521, United States; Department of Statistics, University of California, Riverside, CA 92521, United States; Plant Disease Control Division, National Institute of Agricultural Sciences, Rural Development Administration, Wanju 55365, Korea; US Department of Agriculture-Agricultural Research Service, San Joaquin Valley Agricultural Sciences Center, Parlier, CA 93648, United States; US Department of Agriculture-Agricultural Research Service, San Joaquin Valley Agricultural Sciences Center, Parlier, CA 93648, United States; Department of Botany and Plant Sciences, University of California, Riverside, CA 92521, United States; Institute for Integrative Genome Biology, University of California, Riverside, CA 92521, United States; Department of Plant Pathology and Microbiology, Texas A and M University, College Station, TX 77843, United States; Department of Plant Pathology and Microbiology, Texas A and M University, College Station, TX 77843, United States; Department of Plant Pathology and Microbiology, Texas A and M University, College Station, TX 77843, United States; Center for Phage Technology, Texas A and M University, College Station, TX 77843, United States

**Keywords:** *Xylella fastidiosa*, population genomics, accessory genome, pangenome, prophages, plasmids, carbohydrate-active enzymes, plant-pathogen interactions, lineage diversification

## Abstract

Population structure can shape phenotypic and genomic diversity within microbial pathogens, but how accessory genome variation contributes to diversification below the subspecies level varies across bacterial species. *Xylella fastidiosa* subsp. *multiplex* (*Xfm*) is a xylem-limited plant pathogen with a broad host range, yet the genomic basis of variation among plum-associated lineages remains poorly understood. Here, we combined phenotypic assays with comparative genomics to characterize plum-associated isolates from California and Texas within a broader *Xfm* population framework. The isolates differed in pathogenicity in tobacco, with Texas isolates generally exhibiting greater disease severity than California isolates. Core- and accessory-genome analyses resolved plum-associated isolates into multiple sequence-type-defined lineages rather than a single host-restricted clade. Accessory genome comparisons revealed pronounced lineage-associated structure, with ST26 isolates harboring larger accessory gene repertoires than ST34 isolates and distinct sets of candidate genes associated with mobility, prophage functions, regulation, host interaction, and metabolism. Plasmids were also non-randomly distributed among lineages and comprised conserved family-specific backbones with variable accessory regions and distinct complements of predicted mobility-associated genes. CAZyme repertoires were largely conserved, whereas GH24 showed substantial copy-number and sequence variation within and among isolates, with higher copy numbers particularly evident in ST26 backgrounds. Prophage repertoires were highly heterogeneous and modular, comprising both broadly shared and lineage-restricted clusters that contributed additional structure to the accessory genome. Together, these findings show that plum-associated *Xfm* comprises genomically distinct lineages in which variation in plasmids, prophages, accessory genes, and GH24 repertoires contributes to genomic diversification and aligns with observed differences in pathogenicity.

## Introduction

Plum leaf scald is a chronic and economically significant disease of stone fruit caused by *Xylella fastidiosa*, a xylem-limited, insect-transmitted bacterium responsible for diseases across numerous perennial crops [1–4]. Once established, *X. fastidiosa* infections are difficult to manage because of systemic vascular colonization, and current management relies largely on vector suppression and removal of infected plants [3, 5]. Plum-infecting strains are predominantly associated with *X. fastidiosa* subsp. *multiplex* (*Xfm*), a subspecies characterized by a broad host range and substantial genetic diversity [4, 6]. Variation in disease expression among plum-associated populations [2, 7], however, raises questions about the extent and biological relevance of diversity below the subspecies level.

The association of *X. fastidiosa* with *Prunus* extends beyond cultivated plum and encompasses multiple *Prunus* hosts and bacterial genetic backgrounds. The current EFSA host-plant database documents *X. fastidiosa* detections across diverse *Prunus* species, illustrating the breadth of this host association [8]. Ornamental plum (*Prunus cerasifera*) is also widely distributed and can harbor *X. fastidiosa*, potentially providing epidemiological connectivity between ornamental and agricultural environments [9–12]. At the bacterial population level, plum-associated *Xfm* isolates encompass multiple multilocus sequence types (STs) and geographic origins, indicating that their association with plum is not restricted to a single genetic lineage. This diversity provides an opportunity to examine genomic and phenotypic variation among genetically distinct *Xfm* populations associated with a common host context.

*Xylella fastidiosa* subspecies and strain delineation has traditionally relied on multilocus sequence typing (MLST) and core-genome phylogeny [13, 14], but increasing evidence indicates substantial genomic variation below these taxonomic levels. Strain-level differences can influence host compatibility, persistence, and disease expression [15, 16], highlighting the value of higher-resolution genomic approaches for resolving population structure and identifying genomic features associated with phenotypic variation. Comparisons among plum-associated isolates representing distinct genetic backgrounds therefore provide a framework for determining whether phenotypic variation corresponds with broader differences in genomic composition, while recognizing that ST, host origin, and geography may not be independently represented within available isolate collections. The accessory genome, including plasmids, genomic islands, and prophages, represents an important source of bacterial diversification through gene gain, loss, and recombination [17–21]. Accessory-genome variation occurs even among closely related *X. fastidiosa* strains [22, 23], yet its organization among plum-associated *Xfm* lineages remains poorly resolved. Plasmids and prophages are particularly dynamic components of genome plasticity [23–28] and may provide additional resolution beyond relationships inferred from the conserved chromosome.

Variation in genes associated with metabolism and host interaction represents another potential axis of genomic differentiation. Although *X. fastidiosa* encodes a relatively limited repertoire of carbohydrate-active enzymes (CAZymes) consistent with its xylem-associated lifestyle [29–31], variation in gene composition or copy number occurs among genomic backgrounds and may influence host-associated functions [32]. Prophages can likewise contribute to genomic and regulatory variation [25, 26, 33–37], providing an additional genomic compartment through which diversity among closely related populations can be examined.

Here, we integrate phenotypic characterization with comparative genomics to investigate diversity among plum-associated *Xfm* isolates representing distinct STs and geographic origins within the context of broader *Xfm* diversity. We combine surrogate-host pathogenicity assays with genome-scale analyses to (i) resolve core- and accessory-genome relationships among plum-associated isolates, (ii) characterize the distribution and organization of mobile genetic elements, including plasmids and prophages, and (iii) evaluate variation in CAZyme repertoires across genomic backgrounds. Together, these analyses characterize multiple levels of lineage-associated genomic variation accompanied by differences in pathogenicity among the plum-associated isolates examined and provide a framework for understanding diversification below the subspecies level.

## Materials and methods

### Bacterial strains, isolation, and culture conditions

Plum-associated *Xfm* isolates were recovered from symptomatic trees exhibiting leaf scorch, marginal necrosis, and canopy decline. An initial collection yielded three isolates using a previously described standardized culture-based workflow [4]. Subsequent collections in 2023 and 2025 yielded eight and four additional isolates, respectively, from ornamental *P. cerasifera* in California and agricultural *Prunus salicina* (“Methley”) in Texas. Of the 15 isolates recovered across these collections, nine yielded sufficient high-quality genomic DNA for whole-genome sequencing and comparative genomic analyses. In addition, the previously characterized isolate Riv5 (GCF_015475955.1) was re-sequenced, resulting in a total of 10 isolates sequenced in this study. Symptomatic petioles and stems were surface sterilized with 70% ethanol and rinsed with sterile water. Xylem sap was expressed and plated onto buffered charcoal yeast extract (BCYE) or periwinkle wilt medium (PWM), followed by incubation at 28°C for 21–30 days. Colonies exhibiting morphology consistent with *X. fastidiosa* were purified by repeated streaking, maintained on PWM agar, and archived at −80°C in glycerol stocks. For subsequent experiments, cultures were grown on PWM agar or in PWM broth at 28°C, with liquid cultures shaken at 120 rpm. Low-passage cultures were used throughout to minimize phenotypic drift. Metadata for all newly sequenced and publicly available genomes included in the study are provided in Supplementary Table S1.

### Greenhouse pathogenicity assays

Pathogenicity assays were performed using tobacco (*Nicotiana tabacum* cv. Petit Havana SR1) as a surrogate host. Plants were grown under controlled greenhouse conditions (25–28°C day/20–22°C night; 16-h photoperiod; 50%–60% relative humidity) to the 4–6-leaf stage. Bacterial inocula were prepared from PWM agar cultures, resuspended in PBS, and adjusted to OD_600_ = 0.3–0.5 (~10^8^ CFU ml^−1^). Plants were inoculated by stem puncture at three sites using 50 μl per site, while mock-inoculated controls received PBS. Plants were arranged in a randomized complete block design. Disease severity was evaluated weekly using a 0–5 scale: 0, no visible leaf scorch; 1, 1%–20% of foliage showing yellowing along leaf-tip margins; 2, 21%–40% showing marginal yellowing progressing to scorch; 3, 41%–60% showing leaf-tip scorch with a surrounding yellow halo; 4, 61%–80% showing scorch, with yellowing affecting more than half of the leaves; and 5, 81%–100% showing severe scorch with necrosis [38]. Each plant received one composite whole-plant score per assessment, and scoring was performed blind to isolate identity. Three independent experiments were conducted, each comprising five biological replicate plants per isolate. Weekly severity measurements from each plant were used to calculate individual plant-level AUDPC values, which were subsequently analyzed across experiments. Thus, each treatment comprised 15 plants overall.

### Whole-genome sequencing, assembly, annotation, and public genome standardization

Genomic DNA was extracted using the DNeasy Blood and Tissue Kit (Qiagen), verified by gel electrophoresis, and quantified fluorometrically. Illumina paired-end and Oxford Nanopore long-read libraries were generated, with Illumina sequencing providing >100× coverage. Illumina reads were quality-filtered using fastp v0.23.2, and Nanopore reads were processed prior to assembly. Hybrid genome assemblies were generated using Unicycler v0.5.0, followed by assessment of assembly contiguity and circularity and short-read polishing [39, 40]. Assembly completeness was evaluated using BUSCO v6.0.0. To ensure consistent gene prediction across the dataset, newly sequenced genomes and publicly available *Xfm* assemblies were annotated or re-annotated using Bakta v1.5.1 [41]. Low-quality and duplicate publicly available assemblies were excluded from downstream comparative analyses.

### Genome dataset acquisition and quality control

A total of 278 *X. fastidiosa* subsp. *multiplex* assembly records were retrieved from NCBI on 31 May 2025. Duplicate records, defined as multiple GCA/GCF entries or assembly versions representing the same strain, were resolved by retaining one representative based on current RefSeq status, assembly level, contig number, and N50 (Table S1). Assemblies were retained if they had an expected genome size of approximately 2.5 Mb, N50 ≥ 50 kb, CheckM2 completeness ≥95%, CheckM2 contamination ≤2%, and complete Bakta annotation outputs. Of the 278 records, 110 redundant or superseded assemblies and 84 assemblies that failed one or more quality criteria were excluded. An additional 45 records were removed during diversity-based selection or because of insufficient metadata, resulting in 39 public genomes. These were combined with 10 genomes from isolates sequenced or re-sequenced in this study, including Riv5, yielding a final dataset of 49 genomes. All genomes were re-annotated using Bakta to standardize gene prediction and annotation before downstream comparative analyses (Table S1).

### Pangenome construction and phylogenetic analyses

Annotated genomes were analyzed using Roary v3.13.0 with a protein identity threshold of 80% [17, 40–42]. Gene families were classified as core (≥99% of genomes), soft core (95–<99%), shell (15–<95%), or cloud (<15%) according to their distribution across the dataset. The 80% identity threshold was selected based on its previous use in microbial pangenome analyses, including comparative genomic studies of *X. fastidiosa*. To assess the sensitivity of pangenome composition to the ortholog-clustering threshold, the analysis was repeated at 90% and 100% protein identity using the same 49 genomes and otherwise identical settings. Core genes were concatenated and aligned using MAFFT v7.490 [43]. Core-genome phylogenies were reconstructed using IQ-TREE v2.2.2.6 under the GTR + F + R4 model with 1000 ultrafast bootstrap replicates and SH-aLRT support [44], and trees were visualized using FigTree [45]. Accessory-genome relationships were assessed from gene presence–absence profiles, and pangenome dynamics were evaluated using gene accumulation curves. Topological congruence between the core- and accessory-genome trees was quantified using Robinson–Foulds (RF) distance implemented in the Phangorn R package [46]. Both trees contained the same 49 taxa and were compared as unrooted trees using raw and normalized RF distances. To account for the potential influence of homologous recombination on core-genome phylogenetic inference, recombinant regions were identified and filtered from the concatenated core-genome alignment using Gubbins v3.3.5 [47]. A recombination-filtered phylogeny was then reconstructed from the filtered polymorphic-site alignment using IQ-TREE v2.2.2.6 under the GTR model with gamma-distributed rate heterogeneity and 1000 support replicates. The resulting topology was compared with the unfiltered core-genome phylogeny to evaluate the influence of recombination on major lineage relationships.

### Lineage-associated functional analyses

Lineage-associated functional features were evaluated separately using KEGG Orthology (KO) and PFAM presence–absence matrices derived from eggNOG annotations. Analyses were restricted to STs represented by at least three genomes. For each functional feature, prevalence within the focal ST was compared with prevalence among all remaining genomes using Fisher’s exact test. Effect sizes were summarized using odds ratios and prevalence differences, and *P* values were adjusted for multiple testing using the Benjamini–Hochberg procedure. Features were classified as descriptive lineage-associated candidates when they occurred in ≥60% of genomes within the focal ST, ≤40% of the remaining genomes, and had a prevalence difference ≥ 0.40. Features meeting the prevalence-difference criterion and a Benjamini–Hochberg-adjusted *P* ≤ 0.05 were considered statistically supported lineage-associated features.

### Plasmid identification, classification, phylogeny, and gene synteny analyses

Plasmids were identified based on contig circularity, size, BLASTn similarity, and the presence of replication- and mobility-associated genes. Initial size-based groupings were subsequently evaluated using nucleotide sequence similarity, sequence coverage, and conserved synteny with characterized *X. fastidiosa* reference plasmids. This analysis resolved five sequence-supported families: pXF1.3-like (NC_002489.3) [48], pXF26-like (CP090512.1) [49], pXFAS01-like (CP001012.1) [50], pXF51-like (NC_002490.1) [51], and pXF64-HB-like (NZ_CP009886.1) [52]. Plasmids lacking sufficient whole-sequence similarity to these references were excluded from family-specific analyses. Sequences within each plasmid family were aligned using MAFFT, and phylogenies were reconstructed with IQ-TREE under the GTR + F + R4 model. For comparative synteny analyses, plasmid sequences were rotated to shared anchor genes and consistently oriented before visualization with clinker v1.3.20, followed by manual inspection of gene organization. Plasmid presence–absence was mapped across the dataset, with each plasmid treated as a single-copy element. The small pXF1.3-like plasmids were excluded from functional gene-content analyses because of their limited coding capacity.

### CAZyme analysis

CAZymes were identified using dbCAN v13.0 with the CAZy database [53]. High-confidence hits were retained and classified into CAZy families, including glycoside hydrolases (GH), glycosyltransferases (GT), carbohydrate esterases (CE), auxiliary activities (AA), carbohydrate-binding modules (CBM), and polysaccharide lyases (PL). Protein sequences were grouped by family and aligned using MAFFT v7.505. Families represented by fewer than three sequences or lacking sequence variation were excluded from phylogenetic analyses. Phylogenies were reconstructed using IQ-TREE v3.0.1 with model selection and branch-support assessment where applicable. GH24 proteins were analyzed separately to evaluate copy-number and sequence variation across genomic backgrounds. Within-isolate GH24 copies were compared at the predicted protein-sequence level to determine the number of unique variants, identify identical copies, and quantify pairwise sequence identity. A presence–absence and copy-number matrix of CAZyme families was generated for comparative analyses.

### Prophage identification, clustering, distribution analyses, and comparative genome architecture

Prophage regions were identified using Phigaro v2.4.0. Prophage sequences were standardized and clustered using MMseqs2, resulting in 124 clusters (C1–C124). Presence–absence matrices were generated across genomes, and clusters were classified as plum-specific, non-plum-specific, or shared according to their distribution among isolates. Cluster size and mean prophage length were calculated to characterize variation across clusters. Prophage distribution across STs was further evaluated using presence–absence patterns and hierarchical clustering. Representative prophages from selected clusters were annotated using Bakta and compared with clinker to assess conserved genomic modules and variation in gene content and organization.

### Statistical analyses

Pathogenicity data were analyzed using R. Disease progression was evaluated across three independent experiments, each containing five biological replicate plants per treatment. Area under the disease progress curve (AUDPC) was calculated from weekly disease-severity measurements for each plant. Differences in AUDPC among isolates were evaluated using models accounting for experimental trial, followed by Tukey-adjusted pairwise comparisons among bacterial isolates. Comparisons of individual isolates with the PBS control were performed using Dunnett-adjusted tests. Statistical significance was assessed at α = 0.05. For genomic analyses, Fisher’s exact tests with Benjamini–Hochberg correction were used for lineage-associated functional comparisons where applicable. Other genomic patterns were evaluated using phylogenetic relationships, presence–absence matrices, gene-content clustering, and comparative genome architecture as described above. Statistical summaries and figures were generated using R and Python.

### Data availability

Raw sequencing reads have been deposited in the NCBI Sequence Read Archive (SRA) under BioProject PRJNA1449158, with public release scheduled upon publication. Corresponding accession numbers are provided in Table S1 and will become publicly available upon release. Supplementary datasets supporting the comparative genomic analyses, including pangenome outputs, gene presence–absence matrices, CAZyme annotations, plasmid datasets, prophage clustering tables, and representative prophage annotations, have been deposited in the Dryad Digital Repository (click here to access DOI) and are currently under embargo pending publication. Phylogenetic alignments, tree files, and scripts used for statistical analyses and figure generation are also included in the Dryad repository and will be made publicly available upon publication to facilitate reproducibility.

## Results

### Plum-associated isolates exhibit variation in pathogenicity in a tobacco surrogate host

Because *Xfm* can be difficult to recover from woody tissues, where bacterial titers are often low, a standardized isolation workflow [4] was used to obtain viable cultures from symptomatic plum material collected in California and Texas (Fig. S1A). Isolates were recovered from Riverside, California (ST34: OPR27, OPR29, and Riv5), and from multiple locations in Texas, including Mexia (ST26: Tx-467, Tx-468, Tx-470, and Tx-471), Overton (ST26: Tx-2; ST34: Tx-B), and Houston (ST34: Tx-9). Pathogenicity assays using tobacco as a surrogate host revealed significant variation in disease progression among the isolates examined (Fig. 1A). All bacterial isolates produced significantly greater disease severity than the PBS control (Dunnett-adjusted *P* < .0001; Fig. S1B). AUDPC also differed significantly among bacterial isolates (*P* < 1 × 10^−4^; Fig. 1B), with Texas isolates generally exhibiting higher AUDPC values than California isolates across three independent experiments. However, because ST and geographic origin were not independently represented among the isolates examined, the observed geographic pattern cannot be attributed to geography independently of genomic background. Together, these results demonstrate reproducible variation in disease severity among the plum-associated *Xfm* isolates examined.

**Figure 1:**
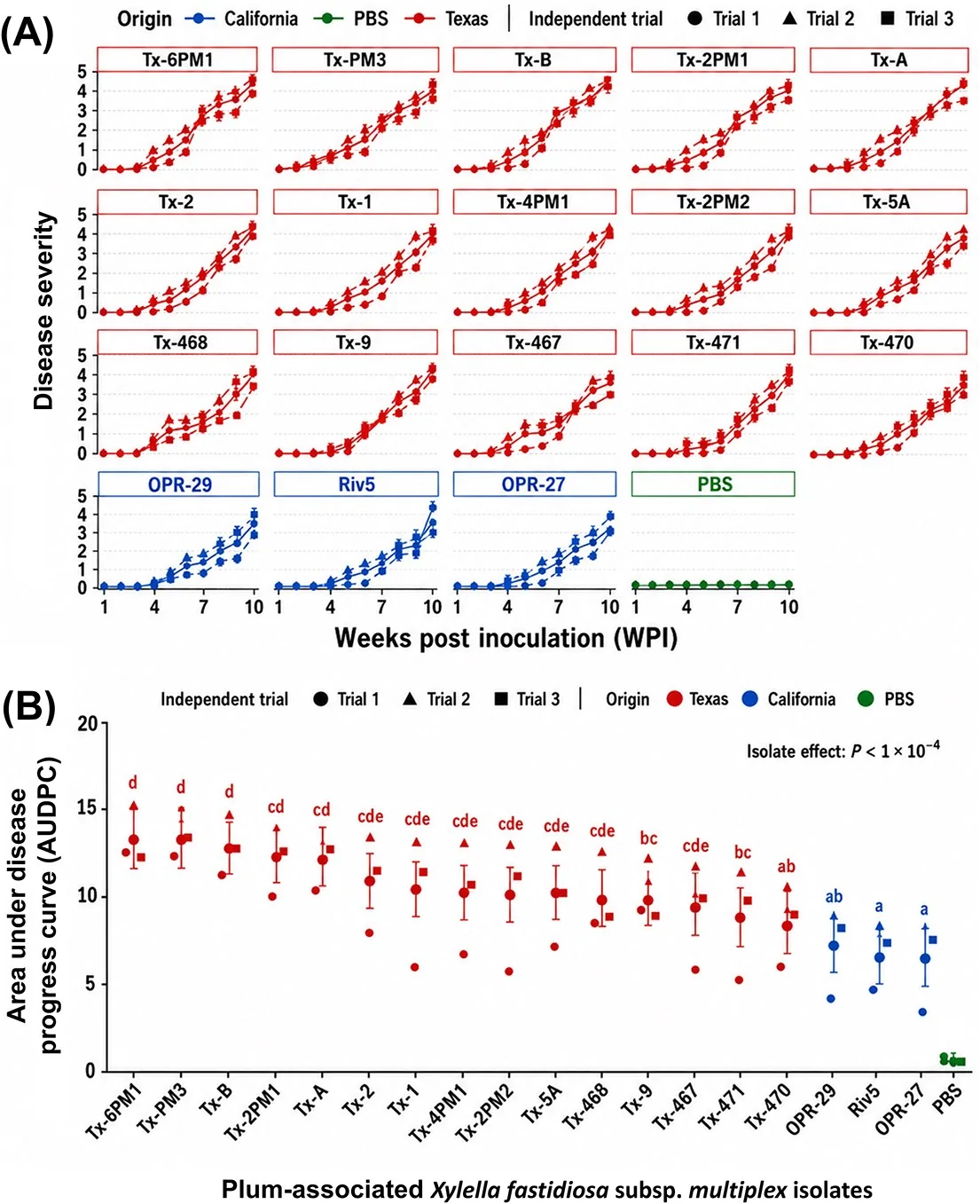
Disease progression and severity of *Xylella fastidiosa* subsp. *multiplex* isolates in tobacco. (A) Disease severity over 10 weeks postinoculation for Texas and California isolates and the PBS control across three independent experiments, each with five replicate plants per treatment. (B) AUDPC values for the three independent experiments; large points and error bars show estimated marginal means and 95% confidence intervals. Different letters indicate Tukey-adjusted differences among bacterial isolates; PBS was excluded from the mean-separation analysis. The isolate effect was significant (*P* < 1 × 10^−4^).

### Core- and accessory-genome analyses reveal lineage-structured population diversity across the global *multiplex* population

The 49-genome dataset contained 1606 core gene families, and the concatenated core-gene alignment comprised 1 545 580 nucleotide sites, including 16 620 variable (1.08%) and 11 512 parsimony-informative (0.74%) sites (Fig. S3B). Core-genome phylogeny showed population structure largely corresponding to ST, with additional patterns related to host and geographic origin (Fig. 2; Table S1). Plum-associated isolates occurred across multiple phylogenetic backgrounds. Isolates sequenced in this study separated principally into ST34 and ST26 groups, while publicly available plum-associated isolates included ST10, ST41, and ST6 (Fig. 2; Table S1). These relationships were largely retained after recombination filtering (Fig. S2). Pangenome analysis showed continued expansion of the total pangenome with genome addition, whereas the core genome approached stabilization (Fig. S3A). Results were similar at the 80% and 90% protein identity thresholds, with 1606 and 1587 core gene families and total pangenomes of 6723 and 6858 gene families, respectively (Table S2). At 100% identity, the core decreased to 987 gene families and the total pangenome increased to 7862 gene families (Table S2). Accessory-gene presence–absence analysis produced a topology distinct from the core-genome phylogeny but retained lineage-associated patterns (Fig. 3). ST34 and ST26 plum-associated isolates formed separate accessory-genome groups, while other plum-associated isolates occurred across multiple genomic backgrounds. Overall, these analyses indicate that plum-associated *Xfm* isolates occur in multiple genetic lineages rather than forming a single plum-associated lineage.

**Figure 2:**
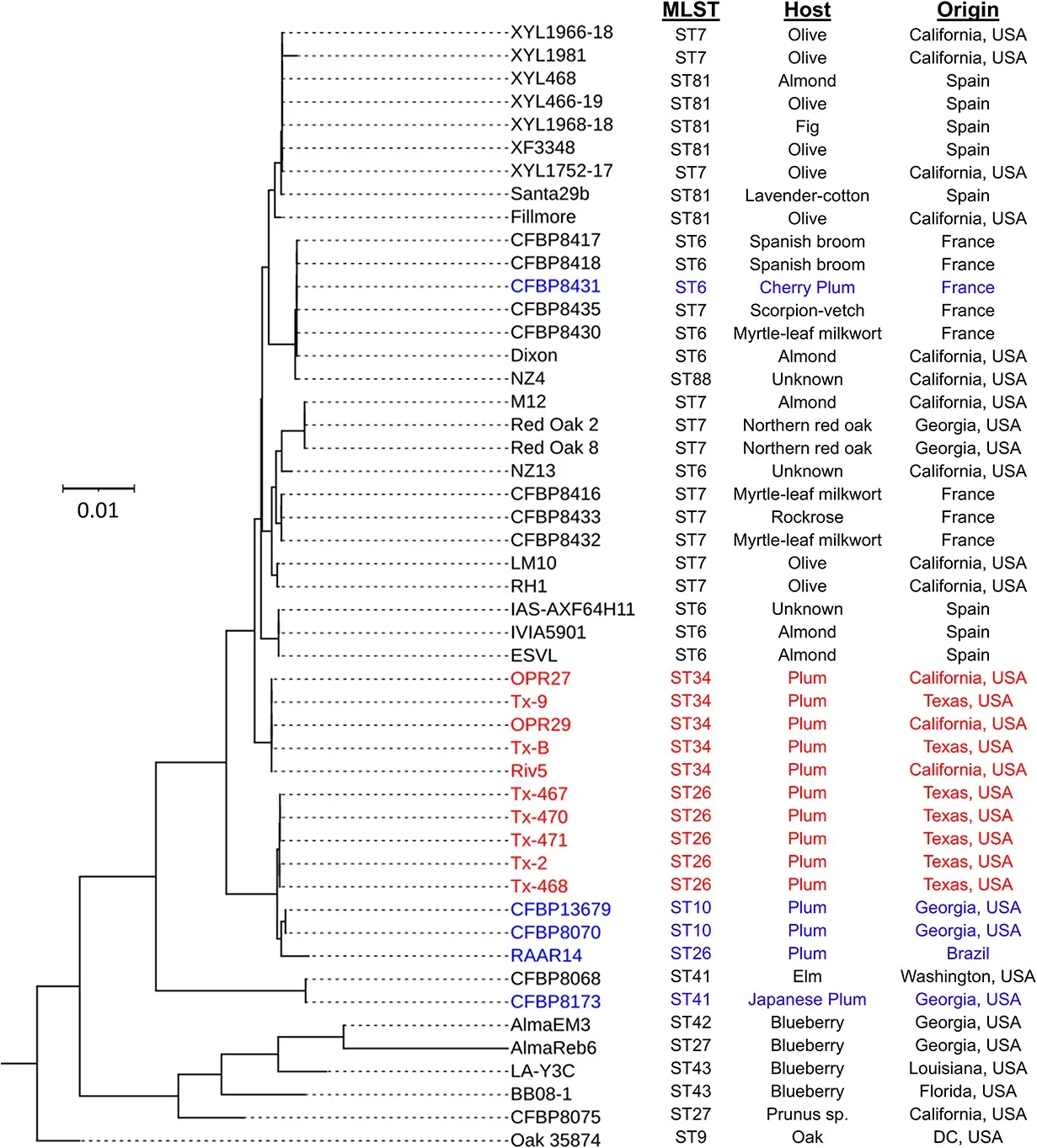
Core-genome phylogeny of global *Xylella fastidiosa* subsp. *multiplex*. Maximum-likelihood phylogeny inferred from concatenated core genes. Isolates sequenced here are shown in red and previously reported plum-associated isolates in blue. MLST, host, and geographic origin are indicated. The phylogeny resolves established lineage structure and positions plum-associated isolates across multiple genetic backgrounds.

**Figure 3:**
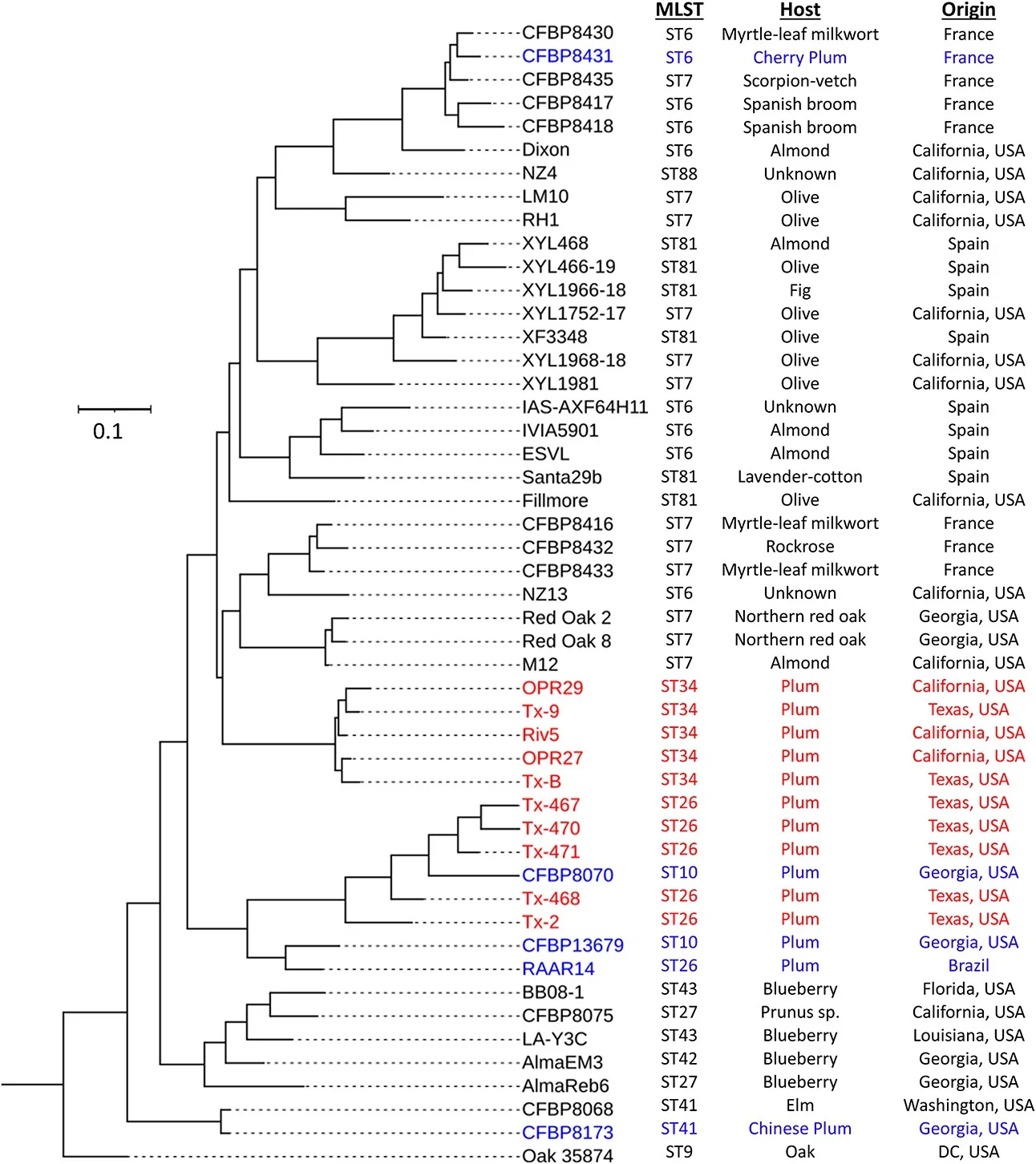
Accessory-genome structure of global *Xylella fastidiosa* subsp. *multiplex*. Maximum-likelihood phylogeny based on gene presence–absence profiles. Isolates sequenced here are shown in red and previously reported plum-associated isolates in blue. The accessory genome resolves gene-content structuring across lineages and delineates two principal plum-associated groups.

### Accessory genome variation identifies lineage-associated functional differentiation and candidate genes in plum-associated *X. fastidiosa*

Accessory gene content varied among plum-associated isolates and showed patterns corresponding to lineage and ST (Fig. 4; Figs S4–S5). ST26 isolates generally shared larger accessory gene sets, including lineage-restricted intersections, whereas ST34 isolates had smaller shared repertoires (Fig. 4). Many accessory genes occurred in limited isolate combinations or were isolate-specific, and poorly characterized genes represented a substantial component of the accessory genome (Figs S4–S5). Functional profiling further identified lineage-associated patterns. Among the six ST26 genomes, eight KEGG-KO and 19 PFAM features met the predefined prevalence-based criteria for descriptive candidates, including functions related to regulation, stress responses, carbohydrate metabolism, and mobile genetic elements; however, none remained significant after Benjamini–Hochberg correction. Among the five ST34 genomes, eight KEGG-KO and nine PFAM features met the descriptive criteria, of which seven KEGG-KO and seven PFAM features remained significant after multiple-testing correction. Several of these features occurred in all ST34 genomes but were absent or infrequent among the remaining genomes. Gene-level comparisons identified additional lineage-associated candidates (Table S3). These represent genes within shared, lineage-restricted accessory gene intersections rather than genes defined by individual isolates. ST26-associated candidates included prophage- and mobility-related loci such as *zot*, *xerD*, phage baseplate-like proteins, and *nicK*, as well as regulatory and stress-associated genes including XRE-family transcriptional regulators, *vapC*, and *yfdQ*. ST34-associated candidates included an RTX toxin, a lipid A biosynthesis-associated acyltransferase, *bglX*, and *gloB*. Overall, these analyses identify differences in accessory gene content and functional composition associated with the major plum-associated STs examined.

**Figure 4:**
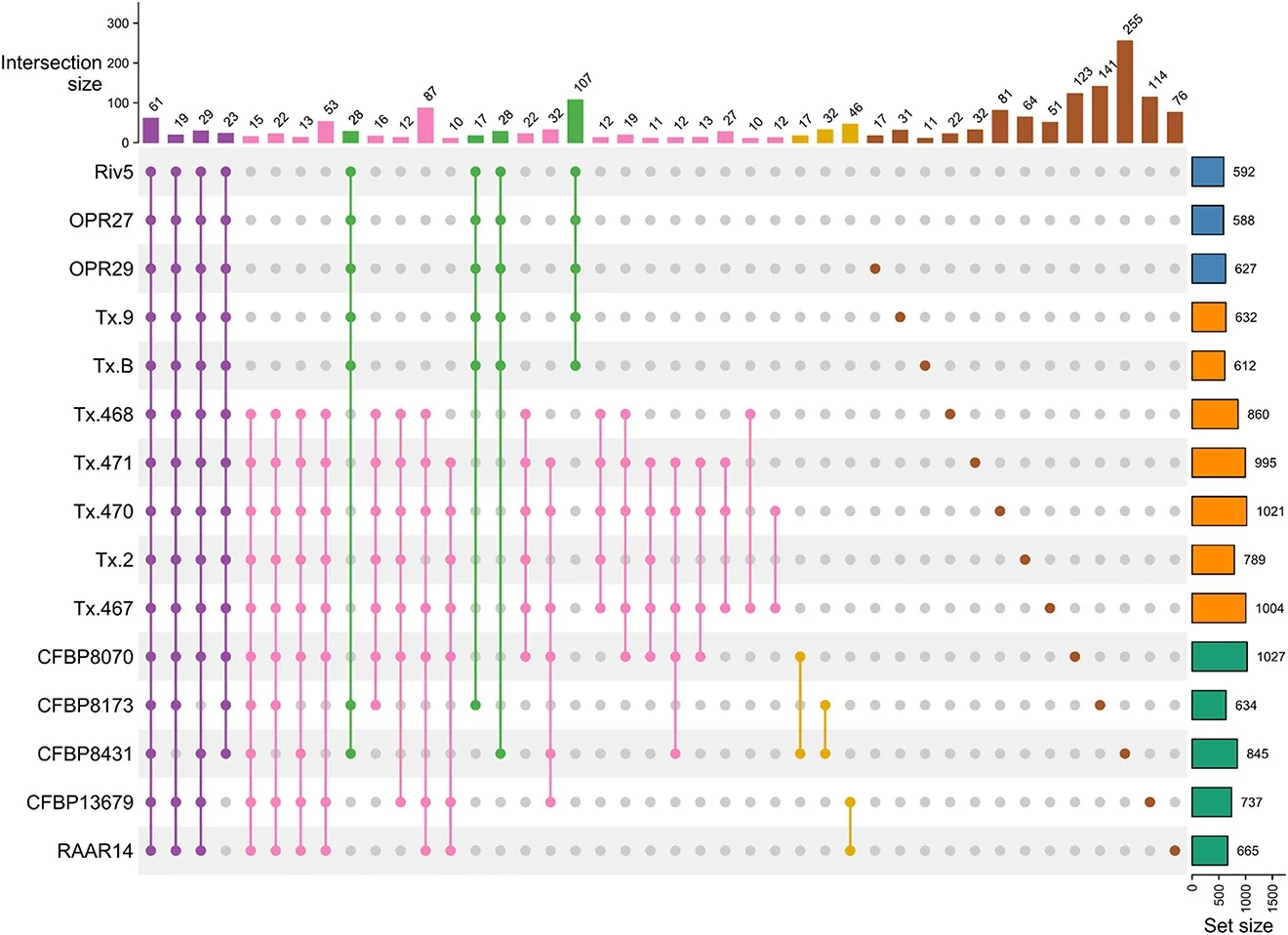
Lineage-structured distribution of accessory genes among plum-associated *Xylella fastidiosa* subsp. *multiplex* isolates. UpSet plot showing the intersection of accessory gene sets across California (blue), Texas (orange), and additional plum isolates (green). Rows correspond to individual isolates, and connected filled circles indicate the specific combination of isolates sharing a given accessory gene set. Vertical bars represent the size of each intersection (number of shared genes), while horizontal bars on the right indicate the total accessory gene count (set size) for each isolate.

### Plasmid distribution and synteny reveal lineage-associated organization with conserved backbones and variable accessory regions

Plasmid carriage across the *Xfm* dataset was limited and showed lineage-associated patterns (Fig. 5; Tables 1–2). Sequence comparisons based on nucleotide identity, coverage, and conserved synteny classified the plasmids into five reference-supported families: pXF1.3-like, pXF26-like, pXFAS01-like, pXF51-like, and pXF64-HB-like (Table 2). Among the 15 plum-associated *Xfm* strains, 13 carried plasmids from one or more of these families, while CFBP8173 (ST41) and CFBP8431 (ST6) lacked detectable homologs (Table 1). pXF51-like and pXF26-like plasmids occurred predominantly in ST10 and ST26 backgrounds, whereas pXFAS01-like plasmids were associated predominantly with ST34 (Fig. 5; Figs S6–S7; Table 2). In contrast, pXF1.3-like plasmids occurred across multiple hosts, STs, and *X. fastidiosa* subspecies (Fig. S8). Within-family comparisons indicated substantial sequence conservation, with nucleotide identities ranging from 83.33%–100% and sequence coverage from 65.82%–100% across the five families (Table 2). Pan-plasmid gene-content analysis broadly recovered the same family structure while identifying variable accessory gene blocks (Fig. S9), and GC content varied within and among families (Fig. S11). Synteny analyses further showed conserved backbones with variable accessory regions (Fig. 6; Fig. S10). pXF51-like plasmids exhibited highly conserved gene order (Fig. 6A), whereas pXF26-like plasmids showed greater structural variation (Fig. 6B). pXFAS01-like plasmids retained a largely conserved backbone with localized variation, while pXF64-HB-like plasmids were extensively collinear (Fig. S10).

**Table 1:** Plasmid profiles of plum-associated *Xylella fastidiosa* subsp. *multiplex* isolates.

Isolate	ST	Region/source	Plum type	Detected plasmid family/families
OPR27	ST34	California	Ornamental plum	pXFAS01-like
OPR29	ST34	California	Ornamental plum	pXFAS01-like
Riv5	ST34	California	Ornamental plum	pXFAS01-like
Tx-9	ST34	Texas	Agricultural plum	pXFAS01-like
Tx-B	ST34	Texas	Agricultural plum	pXFAS01-like
Tx-2	ST26	Texas	Agricultural plum	pXF26-like
Tx-467	ST26	Texas	Agricultural plum	pXF51-like; pXF26-like
Tx-468	ST26	Texas	Agricultural plum	pXF51-like; pXF26-like
Tx-470	ST26	Texas	Agricultural plum	pXF51-like; pXF26-like
Tx-471	ST26	Texas	Agricultural plum	pXF51-like; pXF26-like
CFBP13679	ST10	Georgia	Agricultural plum	pXF51-like; pXF26-like
CFBP8070	ST10	Georgia	Agricultural plum	pXF51-like; pXF1.3-like
CFBP8173	ST41	Georgia	Japanese/Chinese plum	None
CFBP8431	ST6	France	Cherry plum	None
RAAR14	ST26	Brazil	Agricultural plum	pXF51-like; pXF26-like

Distribution of plasmid size classes across isolates, including ST, geographic origin, and host context (ornamental vs agricultural plum). Plasmid sizes are reported as approximate classes, and combinations indicate co-occurrence of multiple plasmids within individual isolates.

**Figure 5:**
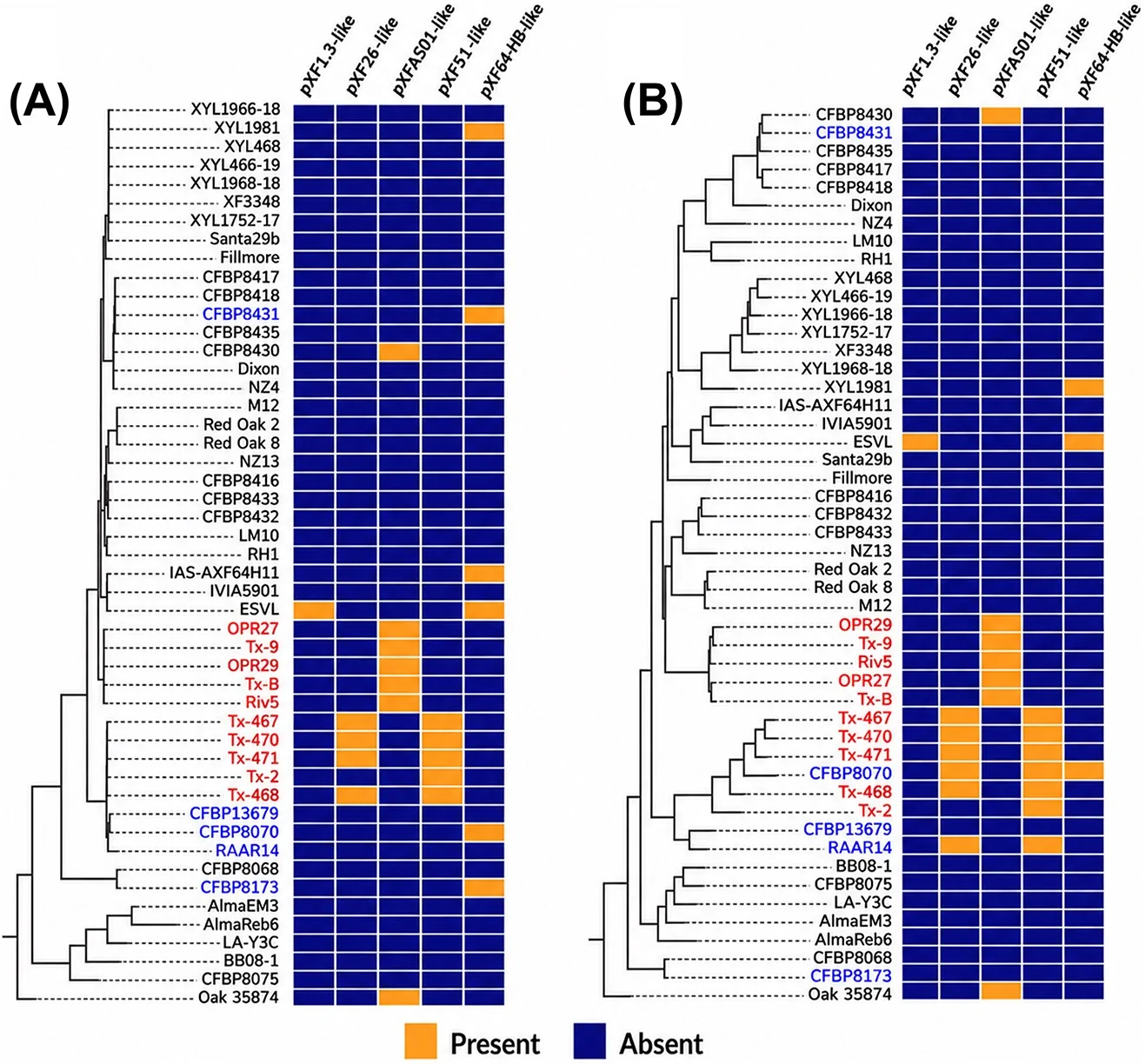
Distribution of plasmid families among *Xylella fastidiosa* subsp. *multiplex* isolates. Core- and accessory-genome phylogenies are shown alongside plasmid presence–absence matrices illustrating the distribution of five plasmid families. Filled and unfilled cells indicate plasmid presence and absence, respectively, highlighting lineage-associated patterns of plasmid distribution.

**Table 2:** Distribution and lineage associations of sequence-defined plasmid families.

Family	n	Size range (kb)	Identity to reference (%)	Coverage (%)	Reference		
					Plasmid used	Accession	Citation
pXF1.3-like	11	1.28–1.38	83.33–100	92.94–100	pXF1.3	NC_002489.3	49
pXF26-like	23	22.60–31.62	93.82–98.55	65.82–100	pXF26-Oak35874	CP090512.1	50
pXFAS01-like	35	35.27–47.86	95.78–98.08	67.49–100	pXFAS01	CP001012.1	51
pXF51-like	11	38.04–51.18	92.51–99.36	79.21–100	pXF51	NC_002490.1	52
pXF64-HB-like	2	59.66–64.25	94.77–99.05	97.69–100	pXF64-HB	NZ_CP009886.1	53

Summary of the major plasmid families identified among *Xylella fastidiosa* subsp. *multiplex* isolates based on nucleotide sequence similarity, coverage, and conserved synteny with characterized reference plasmids. The table summarizes plasmid distribution across STs and highlights conserved, lineage-associated, and rare plasmid families.

**Figure 6:**
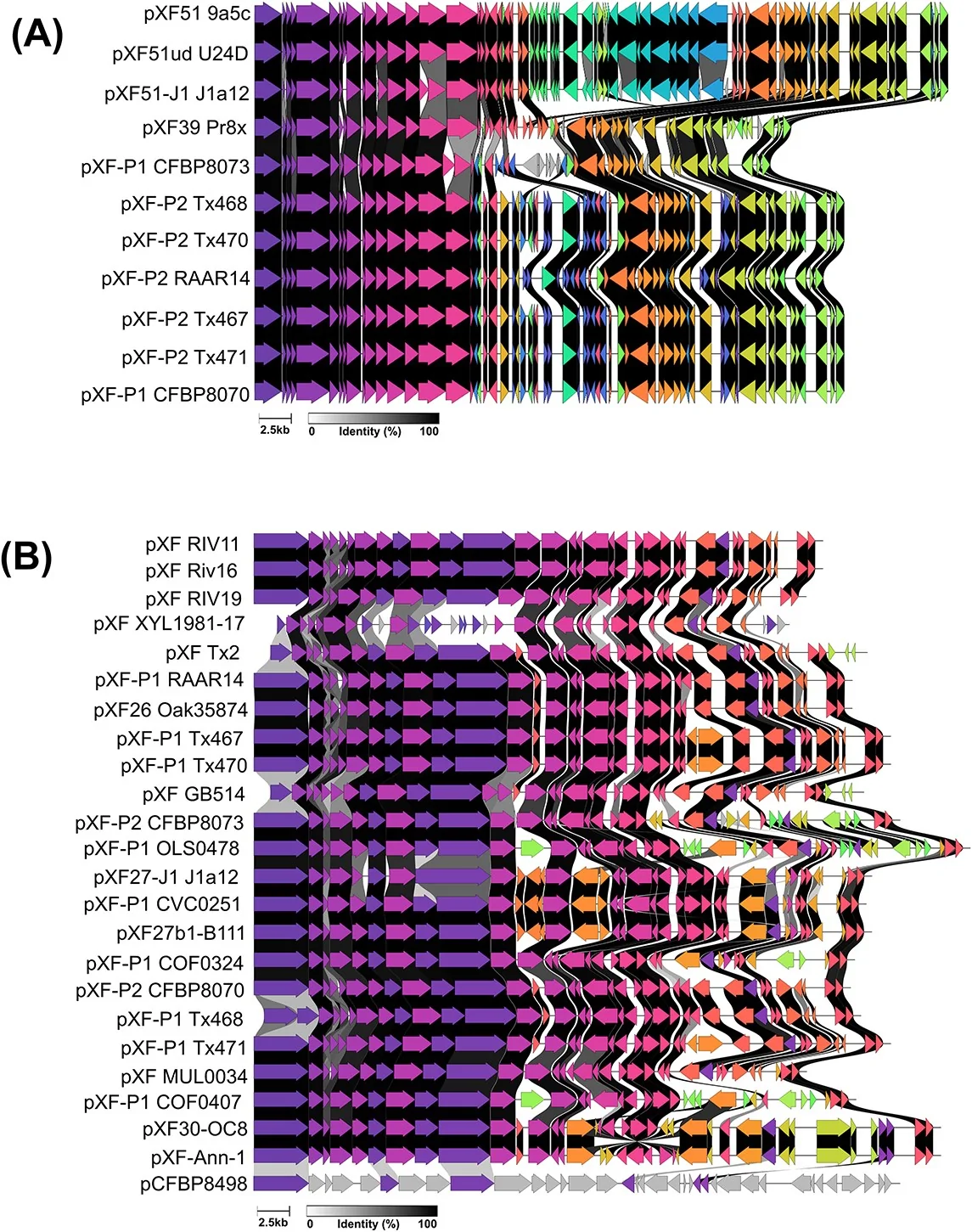
Comparative gene synteny of pXF51-like and pXF26-like plasmids. Comparative gene synteny among 11 pXF51-like plasmids (A) and 23 pXF26-like plasmids (B). pXF51-like plasmids exhibit a highly conserved backbone with localized variation in accessory regions, whereas pXF26-like plasmids show greater structural variation, including differences in gene organization and accessory regions. Arrows represent predicted coding sequences, and shaded links indicate homologous regions and their degree of sequence similarity.

Functional annotation also indicated differences among plasmid families (Figs S12–S15; Table S5). pXFAS01-like plasmids contained a relatively conserved set of *tra* genes together with *virB4* and *virB10*, whereas pXF51-like plasmids encoded multiple *virB* genes and *virD4*. pXF26-like plasmids showed greater variation in *tra*, *virB*, and *virD* genes, while fewer transfer-associated genes were detected in pXF64-HB-like plasmids (Table S5). Predicted mobility-associated gene content varied among plasmid families, although conjugative transfer was not experimentally assessed. BLASTn searches of representative plasmids identified regions similar to plasmids from bacterial taxa outside *Xylella* (Table S6). Representatives of the pXF26-like, pXFAS01-like, pXF51-like, and pXF64-HB-like families showed similarities to plasmid sequences from *Verminephrobacter*, *Xanthomonas*, an uncultured bacterium, and *Paraburkholderia*, respectively, whereas no non-*Xylella* plasmid match was recovered for the pXF1.3-like representative among the retrieved hits. These results indicate broader sequence relationships without establishing horizontal transfer. Overall, the analyses identify plasmid families that differ in lineage distribution, structural organization, and predicted mobility-associated gene content.

### CAZyme repertoires are conserved with lineage-associated GH24 variation across genomic backgrounds

CAZyme analysis showed that most enzyme families were broadly conserved across *Xfm* genomes (Fig. 7A; Fig. S16; Table S9), while a limited subset varied in presence or copy number (Table 3). GH2 occurred in 18% of genomes, whereas GH25 and GH6 occurred in 71% and 80%, respectively (Fig. S16; Tables 3, S9). These families varied across multiple STs, including ST10, ST26, and ST34. In contrast, GH24 showed substantial copy-number variation (Fig. 7B; Table 3), with ST26 isolates generally carrying more copies than ST34 isolates. This distribution corresponded with broader genomic lineage structure, although ST, geography, and host were not independently represented in the dataset. Phylogenetic analyses showed sequence variation among GH24 homologs and their distribution across multiple genomic backgrounds (Figs S17–S18). GH24 sequences from newly sequenced and publicly available genomes were distributed across multiple clades, and ST26 isolates contributed a greater number of sequences, consistent with their higher copy numbers (Fig. S17). Analysis of plum-associated isolates further showed that GH24 copy-number variation included both identical and sequence-distinct protein copies (Fig. S18; Table S10). Across the 15 isolates, 105 GH24 copies represented 35 unique protein sequences. Fourteen isolates contained multiple copies; 10 contained at least one pair of identical protein sequences, whereas four contained only sequence-distinct copies. In addition, 13 identical GH24 protein variants were shared between two or more isolates (Table S10). Thus, variation in GH24 copy number was accompanied by both repeated identical copies and sequence variation within and among isolates. Overall, CAZyme repertoires were largely conserved across *Xfm*, while GH24 copy number and sequence variation differed among genomic backgrounds, particularly ST26 and ST34.

**Figure 7:**
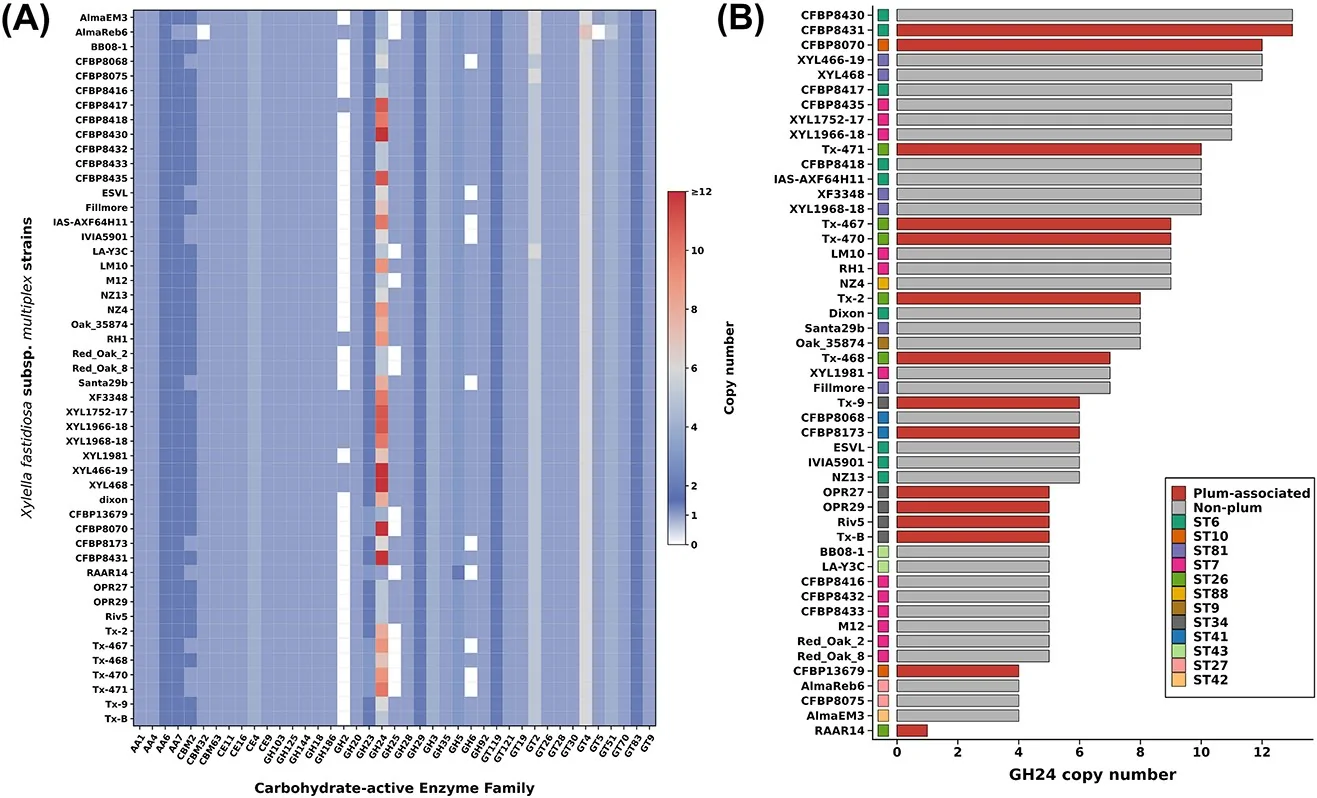
Distribution of carbohydrate-active enzymes (CAZymes) among *Xylella fastidiosa* subsp. *multiplex* isolates. (A) Heatmap showing CAZyme gene copy numbers across isolates, with differences in shading intensity representing variation in copy number. (B) Bar plot comparing GH24 gene copy numbers among isolates, highlighting lineage associated variation in GH24 distribution.

**Table 3:** CAZyme family variation across *Xylella fastidiosa* subsp. *multiplex* genomes.

CAZyme family	Presence/copy-number pattern	Notes
GH2	Infrequent presence	Detected in 18% of genomes
GH6	Variable presence	Detected in 80% of genomes across multiple STs
GH24	Variable copy number and sequence diversity	Copy number varied substantially among isolates and STs, with higher copy numbers generally observed in ST26 than ST34; GH24 copies included both identical and sequence-divergent protein variants
GH25	Variable presence	Detected in 71% of genomes across multiple STs

Selected carbohydrate-active enzyme (CAZyme) families exhibiting variation in presence or copy number across isolates. Most CAZyme families were broadly conserved, whereas GH2, GH6, and GH25 varied in presence and GH24 exhibited pronounced copy-number and sequence variation across genomic backgrounds.

### Prophage repertoires are heterogeneous, modular, and structured by ST

A total of 124 prophage clusters were identified across the *Xfm* dataset, with substantial variation in cluster size and prophage length (Fig. S19; Table 4). A few clusters contained many prophages, whereas most were smaller, and prophage length varied considerably among clusters (Fig. S19A–B). Presence–absence analysis further showed sparse and heterogeneous prophage distributions across strains, with many clusters restricted to individual genomes and a smaller subset shared across multiple lineages (Fig. 8; Fig. S21). CFBP13679, in which no prophage clusters were detected, was excluded from the Jaccard-based hierarchical clustering in Fig. 8. Comparative analysis of representative clusters showed conserved genomic modules alongside variation in gene content, order, and orientation, consistent with modular prophage organization (Fig. S22). In addition to canonical phage-associated functions, prophage regions contained genes annotated with putative regulatory and other accessory functions, although their functional effects were not evaluated in this study. Analysis across STs further showed both broadly distributed and lineage-restricted prophage clusters (Fig. S20; Table S11). C1–C4 occurred across multiple STs, whereas many clusters were restricted to one or a few STs, including clusters associated with plum-derived ST10, ST26, and ST34 backgrounds. Within ST34, broadly distributed clusters such as C44, C41, and C17 occurred alongside the plum-associated C31 cluster (Table S11). Other clusters were largely associated with non-plum ST6, ST7, and ST81 backgrounds, while ST26 and ST34 contained comparatively diverse prophage repertoires (Fig. S20; Table S11). Overall, prophage repertoires in *Xfm* were unevenly distributed and structurally diverse, with both shared and lineage-restricted elements contributing to variation across STs.

**Table 4:** Summary statistics of prophage clusters identified in *Xylella fastidiosa* subsp. *multiplex*.

Cluster statistic	Number of clusters	Percentage (%)
Total prophage clusters	124	100
Singleton clusters	47	37.9
Multi-member clusters	77	62.1
Shared clusters	51	41.1
Plum-associated clusters (total)	26	21.0
Plum-specific clusters^1^	16	12.9
Plum-associated, non-specific clusters^1^	10	8.1

Overview of prophage cluster composition and distribution across the *Xfm* dataset. Plum-specific clusters were detected exclusively in plum-associated isolates, whereas plum-associated clusters occurred in both plum and non-plum isolates but were more frequently represented among plum-associated isolates.

^1^Plum-specific clusters were detected exclusively in plum-associated isolates. Plum-associated, non-specific clusters were detected in both plum and non-plum isolates but occurred more frequently among plum-associated isolates.

**Figure 8:**
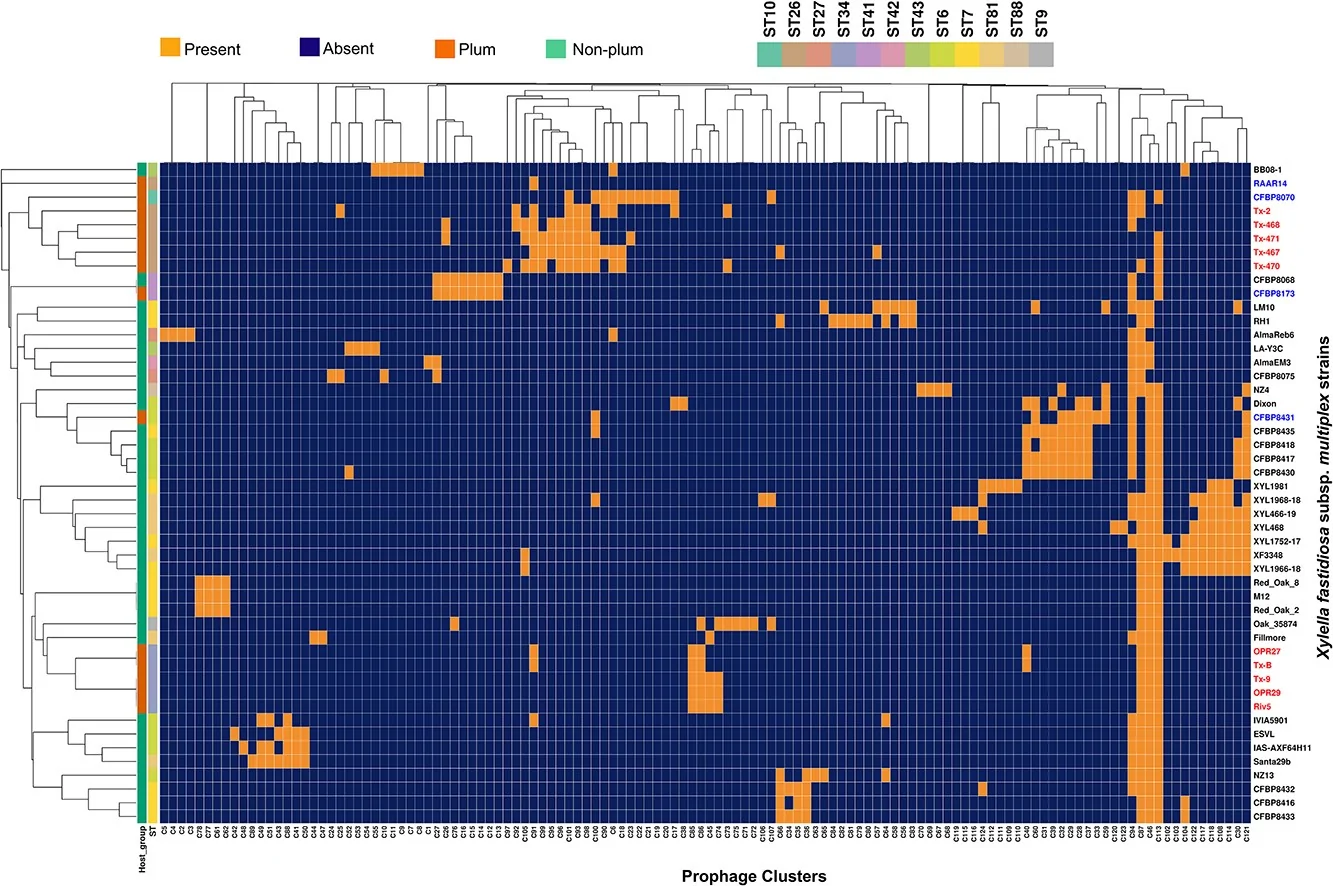
Distribution of prophage clusters among *Xylella fastidiosa* subsp. *multiplex* isolates. Hierarchically clustered heatmap showing the presence and absence of prophage clusters across isolates. Filled and unfilled cells indicate prophage presence and absence, respectively, highlighting variation in prophage content and lineage-associated distribution patterns.

## Discussion

### Pathogenicity and population-genomic structure reflect lineage-associated diversification within subsp. *multiplex*

Plum-associated *Xfm* isolates exhibited variation in pathogenicity in the tobacco surrogate-host assay, with isolates collected from Texas generally producing greater disease severity than those collected from California. Disease development in *X. fastidiosa* is influenced by colonization and persistence within xylem vessels, where surface attachment and biofilm formation contribute to vascular colonization, vessel occlusion, and interactions with insect vectors [54–58]. Although the mechanisms underlying the observed pathogenicity differences were not directly evaluated, the reproducible variation among isolates indicates heterogeneity in disease development under the conditions examined. Importantly, geographic origin, host species, and ST are not independently represented in the current isolate collection and therefore cannot be disentangled. These differences should thus be interpreted as variation among the isolates and lineages examined rather than as independent effects of geography, host, or ST. The population-genomic analyses further showed that plum association occurs across multiple phylogenetic backgrounds. Plum-associated isolates were distributed among ST26, ST34, ST10, ST41, and ST6, indicating that association with *Prunus* is not restricted to a single evolutionary lineage. This pattern distinguishes host association from phylogenetic specialization and is consistent with previous findings that host range in *X. fastidiosa* can reflect ecological compatibility rather than strict lineage-specific adaptation [17, 22, 59].

### Accessory genome variation complements recombination-driven diversification within subsp. *multiplex*

Recombination is recognized as a major contributor to genetic diversification in *X. fastidiosa*, generating variation through the exchange and incorporation of genetic material [60, 61]. Within this established evolutionary framework, the correspondence observed here between core-genome lineages and multiple accessory-genome compartments suggests that accessory-genome variation provides an additional layer of diversity within *Xfm*. Accessory genomes can vary through gene gain and loss, recombination, and turnover of mobile genetic elements [19, 20, 62–64], and previous comparative genomic studies have similarly documented substantial variation in accessory gene content and mobile elements among *X. fastidiosa* strains, with subsp. *multiplex* exhibiting considerable accessory-genome diversity [21, 65]. The lineage-associated organization observed here may therefore reflect differences in the evolutionary histories, acquisition, and retention of accessory components among *Xfm* genomic backgrounds rather than an alternative mechanism to recombination-driven diversification. The functional differences associated with ST26 and ST34 further suggest that distinct lineages maintain different complements of genes related to genome mobility, regulation, metabolism, and interactions with the host environment. These differences may provide alternative genomic configurations through which lineages interact with ecological or host-associated conditions, without necessarily representing lineage-specific adaptations. This interpretation is consistent with the established importance of recombination in *X. fastidiosa* diversification [60, 61], while phylogenetic history remains an important component of accessory-genome organization and host association [22]. The lineage-associated candidate functions identified here therefore provide a basis for investigating how accessory-genome variation contributes to biological differences among *Xfm* lineages. However, their contribution to the observed variation in pathogenicity cannot be inferred from genomic associations alone and will require targeted functional validation.

### Plasmid and prophage compartments reinforce lineage structure through modular and lineage-associated organization

The lineage-associated distribution of plasmids suggests that their persistence within *Xfm* may reflect a combination of horizontal transfer, genomic compatibility, ecological context, and selective retention [66–69]. The presence of *tra/trb* and type IV secretion-associated genes in pXFAS01-like plasmids is consistent with previous characterization of this plasmid lineage, including its occurrence in the plum *Xfm* strain Riv5 [28]. Burbank and Van Horn experimentally demonstrated conjugative transfer mediated by this machinery in *X. fastidiosa*, supporting conjugation as a potential route of horizontal gene transfer. Related plasmids occurring across different *X. fastidiosa* subspecies further provide evidence consistent with horizontal movement of plasmid DNA 58. However, their lineage-associated distributions [23, 24, 28] suggest that mobility potential does not necessarily result in widespread dissemination. Rather, conserved plasmid backbones with variable accessory regions may provide a flexible source of genetic variation whose acquisition and retention differ among genomic backgrounds [21]. Prophages represent a similarly dynamic, but more heterogeneous, component of the accessory genome. Their modular organization and incomplete correspondence with the core-genome phylogeny are consistent with horizontal acquisition, recombination, loss, and modular exchange [17, 23, 34, 37, 50, 70]. Previous studies have similarly implicated prophage-associated regions in genome rearrangement and differentiation in *X. fastidiosa* [37, 71], while the recent genome-wide analysis of Tomás-Tomás *et al.* [72] further demonstrated extensive prophage diversity across the species. Within *multiplex*, the coexistence of broadly shared and lineage-restricted elements likely reflects a combination of inheritance, horizontal acquisition, differential retention, and turnover [73–75]. Predicted regulatory and accessory genes within these regions may provide additional sources of functional variation [37, 71], although their phenotypic consequences remain to be established. Together, plasmids and prophages represent dynamic sources of accessory-genome variation while retaining lineage-associated distributions. These patterns are consistent with the broader view that recombination is a major contributor to diversification in *X. fastidiosa*, with mobile genetic elements providing additional genetic variation shaped by lineage history, horizontal acquisition, recombination, loss, and selection.

### Conserved CAZyme repertoires with lineage-associated GH24 variation highlight gene dosage as a potential axis of differentiation

The broad conservation of CAZyme repertoires across *Xfm* is consistent with the specialized xylem-limited lifestyle of *X. fastidiosa* [1, 23, 76, 77] and with shared requirements for carbohydrate processing and host colonization. Several of these functions contribute to modification of plant cell-wall components and movement through xylem tissues, including enzymes delivered through the type II secretion system [29, 31, 57, 78, 79]. Against this relatively conserved background, variation in GH24 copy number and sequence suggests a more dynamic evolutionary history for this family. GH24 proteins include peptidoglycan-degrading lysozymes involved in bacterial cell-wall remodeling as well as phage-associated lytic functions [80–82]. Their widespread occurrence in phages further supports a potential connection between GH24 diversity and mobile genetic elements [83]. The occurrence of both identical and sequence-divergent GH24 copies within *Xfm* suggests that copy-number variation may reflect a combination of duplication, diversification, differential retention, and potentially acquisition rather than simple expansion of a single allele [84, 85]. This distinction is biologically relevant because additional copies could provide opportunities for functional redundancy or subsequent sequence divergence, although such consequences cannot be inferred from sequence data alone. Previous comparative analyses have also identified lysozyme genes within *X. fastidiosa* prophage regions [21], providing a plausible genomic context for some of this variation. However, the present analyses do not establish a direct relationship between prophages and GH24 expansion. Overall, GH24 variation represents a lineage-associated component of an otherwise relatively conserved CAZyme repertoire and provides a useful target for investigating the evolutionary and functional consequences of gene copy-number variation in *Xfm*.

## Conclusion, limitations, and future directions

Plum-associated *Xfm* isolates exhibit genomic diversity below the subspecies level, with core-genome lineage structure accompanied by variation in accessory gene content, plasmids, prophages, and gene copy number. Their distribution across multiple phylogenetic lineages further indicates that association with *Prunus* is not restricted to a single evolutionary background. While diversification and subspeciation in *X. fastidiosa* have been strongly linked to homologous recombination [61, 86], the present findings suggest that lineage-associated accessory-genome variation provides an additional layer of differentiation within subsp. *multiplex*. Thus, mobile elements and variable gene content may contribute to genomic diversity within the broader framework of recombination-driven evolution.

Several limitations should be considered when interpreting these findings. ST, geographic origin, and host species are partially confounded in the current isolate collection, preventing their independent contributions from being resolved. The isolates were also obtained from two *Prunus* species, further limiting attribution of observed differences specifically to lineage, geography, or host. In addition, pathogenicity was evaluated in tobacco as a surrogate host and therefore may not fully reflect disease responses in plum. *Xylella fastidiosa* infection and bacterial population sizes were not confirmed by re-isolation or molecular quantification at the end of the experiments; consequently, differences in disease development cannot be directly linked to differences in bacterial colonization. The number and geographic representation of available isolates and the lack of functional validation of lineage-associated candidate genes provide additional limitations. Future studies incorporating broader and more balanced sampling, cross-inoculation experiments in relevant hosts, bacterial population measurements, and targeted functional analyses will help determine how lineage, accessory-genome variation, and host context contribute to phenotypic diversity and disease development. Overall, this study provides a population-genomic framework for understanding differentiation among plum-associated *X. fastidiosa* subsp. *multiplex* and identifies accessory-genome features for further investigation.

## Supplementary Material

Supplementary_Materials_ycag291
