# Supplementary material for: Accessory genome dynamics shape lineage-associated diversification in plum-associated Xylella fastidiosa subsp. multiplex
Source: ISME Commun. 2026 Oct 13;6(1):ycag291. doi: 10.1093/ismeco/ycag291 (PMC13755354; doi:10.1093/ismeco/ycag291)
Supplement: Supplementary file 1 [file supplementary_materials_ycag291.zip › Supplementary_Materials_ycag291/Supplementary_figures_xfm-plum_ISMEC_revision1_FINAL_ycag291.docx]

**Figure S1. Geographic distribution and pathogenicity of plum-associated *Xylella fastidiosa* subsp. *multiplex* isolates.** **(A)** Geographic origin of isolates included in the study, showing isolates collected from ornamental plum in Riverside, California, and agricultural plum from multiple locations in Texas (Mexia, Overton, and Houston). Individual isolate identifiers are indicated at their respective collection locations. **(B)** Estimated differences in area under the disease progress curve (AUDPC) for each bacterial isolate relative to the PBS control. Points represent estimated mean differences and horizontal bars represent Dunnett-adjusted 95% confidence intervals. The dashed vertical line at zero indicates no difference from PBS. All bacterial isolates produced significantly greater AUDPC than PBS (Dunnett-adjusted *P* < 0.0001). Isolates are colored according to geographic origin (Texas, red; California, blue).

**Figure S2. Recombination-filtered core-genome phylogeny of *Xylella fastidiosa* subsp. *multiplex*.** Phylogenetic relationships among 49 *X. fastidiosa* subsp. *multiplex* genomes reconstructed after identification and removal of recombinant regions from the core-genome alignment. Sequence types (STs) are indicated adjacent to isolate names. Red indicates plum-associated isolates sequenced in this study, whereas blue indicates publicly available plum-associated isolates included for comparative analysis. The scale bar represents nucleotide substitutions per site.

**Figure S3. Pangenome dynamics and core-genome alignment characteristics of *Xylella fastidiosa* subsp. *multiplex*.** **(A)** Accumulation of pangenome, accessory, core, shell, and cloud gene families across the 49-genome dataset. Curves show changes in gene-family number as genomes were sequentially added across random permutations, with error bars indicating variation among permutations. Continued expansion of the total pangenome and accessory gene fractions with increasing genome number is consistent with an open pangenome. **(B)** Composition of the concatenated core-genome alignment used for phylogenetic reconstruction. The alignment contained 1,545,580 nucleotide sites, including 1,528,960 constant sites (98.92%), 16,620 variable sites (1.08%), and 11,512 parsimony-informative sites (0.74%). Parsimony-informative sites represent a subset of the variable sites.

**Figure S4. Functional category distribution across accessory gene combinations in plum-associated *Xylella fastidiosa* subsp. *multiplex*.** Heatmap showing the proportion (%) of genes assigned to COG functional categories across accessory gene combinations identified in the pangenome analysis. Rows represent individual gene combinations (as defined in the UpSet analysis), and columns correspond to COG categories grouped into major functional classes, including cellular processes and signaling, metabolism, information storage and processing, and poorly characterized functions.

**Figure S5. Functional composition of accessory genomes across lineage groupings in plum-associated *Xylella fastidiosa* subsp. *multiplex*.** Heatmap showing the proportion (%) of genes assigned to COG functional categories across major lineage groupings, including California (CA), Texas (TX), shared (“All”), and unique gene sets. Columns correspond to individual COG categories, grouped into higher-order functional classes (cellular processes and signaling, metabolism, information storage and processing, and poorly characterized functions), and color intensity reflects the relative proportion of each category within a grouping.

**Figure S6. Phylogenetic relationships of pXF51-like and pXF26-like plasmids detected in plum-associated *Xylella fastidiosa* subsp. *multiplex* isolates.** (A) Maximum-likelihood phylogeny of pXF51-like plasmids. (B) Maximum-likelihood phylogeny of pXF26-like plasmids. Trees were reconstructed from plasmid nucleotide alignments using IQ-TREE. Plum isolates sequenced in this study are shown in red, previously reported plum isolates retrieved from NCBI are shown in blue, and the remaining *X. fastidiosa* strains representing other hosts and subspecies are shown in black. Sequence type (ST), host plant, and geographic origin are indicated next to each strain. Both plasmid families were detected among plum-associated *X. fastidiosa* subsp. *multiplex* isolates belonging predominantly to ST10 and ST26.

**Figure S7. Maximum-likelihood phylogeny of pXFAS01-like plasmids.** The tree was reconstructed from plasmid nucleotide alignments using IQ-TREE. Plum isolates sequenced in this study are shown in red, previously reported plum isolates retrieved from NCBI are shown in blue, and the remaining *X. fastidiosa* strains are shown in black. Sequence type (ST), host plant, and geographic origin are indicated next to each strain. Among the plum-associated isolates, pXFAS01-like plasmids were associated predominantly with the ST34 lineage, including California isolates Riv5, OPR27, and OPR29 and Texas isolates Tx-B and Tx-9.

**Figure S8. Phylogenetic relationships of pXF1.3-like plasmids across *Xylella fastidiosa*.** Maximum-likelihood phylogeny of pXF1.3-like plasmid nucleotide sequences. Homologous plasmids were identified based on sequence similarity to the plasmid detected in the plum-associated isolate CFBP8070 and were restricted to small plasmids of comparable size (~1.2–1.3 kb). The CFBP8070 plasmid is highlighted in blue. The phylogeny shows that pXF1.3-like plasmids occur across diverse *X. fastidiosa* subspecies, hosts, geographic origins, and sequence types.

**Figure S9. Pan-plasmid gene presence–absence across the *Xylella fastidiosa* species complex.** Heatmap showing the distribution of orthologous gene clusters identified from pangenome analysis of plasmids across the *X. fastidiosa* species complex. Rows represent individual plasmid sequences and columns represent orthologous gene clusters. Presence (light orange) and absence (navy blue) are indicated for each gene cluster. Hierarchical clustering based on gene content is shown for both plasmids and gene clusters. Row annotations indicate subspecies, host of isolation (plum versus other hosts), and multilocus sequence type (MLST). Plasmids form discrete gene-content clusters broadly corresponding to the sequence-supported plasmid families, while variable gene blocks reflect differences in accessory content among plasmids.

**Figure S10. Comparative gene synteny of pXFAS01-like and pXF64-HB-like plasmids.** Comparative gene synteny among 35 pXFAS01-like plasmids (A) and 2 pXF64-HB-like plasmids (B). pXFAS01-like plasmids show a largely conserved backbone with localized variation in accessory regions, whereas pXF64-HB-like plasmids exhibit extensive collinearity across their length. Arrows represent predicted coding sequences, and shaded links indicate homologous regions and their degree of sequence similarity.

**Figure S11. GC content variation among sequence-supported plasmid families.** GC content of plasmids grouped into pXF1.3-like, pXF26-like, pXFAS01-like, pXF51-like, and pXF64-HB-like families. Individual plasmids are shown within each family, illustrating variation in GC composition within and among plasmid families.

**Figure S12. Functional composition of pXF26-like plasmids.** Proportional distribution of predicted gene functions across pXF26-like plasmids. Mobility-related genes constitute a major functional component, with variable contributions from replication, defense, metabolism, and other functional categories. A substantial fraction of predicted genes remains functionally uncharacterized.

**Figure S13. Functional composition of pXFAS01-like plasmids.** Proportional distribution of predicted gene functions across pXFAS01-like plasmids. Functional profiles show substantial conservation, with mobility-related genes representing a major component and additional contributions from other functional categories. A substantial fraction of predicted genes remains functionally uncharacterized.

**Figure S14. Functional composition of pXF51-like plasmids.** Proportional distribution of predicted gene functions across pXF51-like plasmids. Mobility-related genes are prevalent, together with replication, defense, and other functional components. Variation among plasmids reflects differences in accessory gene content, while a substantial fraction of predicted genes remains functionally uncharacterized.

**Figure S15. Functional composition of pXF64-HB-like plasmids.** Proportional distribution of predicted gene functions across pXF64-HB-like plasmids. Functional profiles are highly similar between plasmids, consistent with the strong conservation of gene content and overall organization observed within this family.

**Figure S16. Distribution of CAZyme families across *Xylella fastidiosa* subsp. *multiplex*.** Presence–absence heatmap of CAZyme families across genomes. Most families are broadly conserved, whereas GH2, GH25, and GH6 show variable presence across the dataset.

**Figure S17. Phylogeny of GH24 proteins across *Xylella fastidiosa* subsp. *multiplex*.** Maximum-likelihood phylogeny of GH24 protein sequences. Proteins from isolates sequenced in this study are shown in red and those from publicly available genomes in blue. Clustering patterns illustrate sequence variation among GH24 homologs and their distribution across different genomic lineages.

**Figure S18. Phylogenetic relationships of GH24 genes across plum-associated *Xylella fastidiosa* subsp. *multiplex* strains.** Circular phylogenetic tree of GH24 gene sequences across plum-associated *X. fastidiosa* subsp. *multiplex* strains. Branch tips are colored by strain identity, and background shading denotes Texas and California plum-associated groups and isolates from other hosts. The tree illustrates sequence variation among GH24 copies and their distribution across different genomic backgrounds.

**Figure S19. Distribution and size characteristics of prophage clusters across *X. fastidiosa* genomes.** (A) Prophage cluster size distribution, showing the number of prophages within each cluster (C1–C124), ranked from largest to smallest. Bars are colored by category: plum-specific (red), shared (gray), and non-plum-specific (blue). (B) Mean prophage length for each cluster, ordered consistently with panel (A). Substantial variation is observed in both cluster size and prophage length, reflecting heterogeneity in prophage composition across clusters and host-associated groups.

**Figure 13. Distribution and size characteristics of prophage clusters across Xylella fastidiosa genomes. (A) Prophage cluster size distribution, showing the number of prophages within each cluster (C1–C124), ranked from largest to smallest. Bars are colored by category: plum-specific (red), shared (gray), and non-plum-specific (blue). (B) Mean prophage length for each cluster, ordered consistently with panel (A). Substantial variation is observed in both cluster size and prophage length, reflecting heterogeneity in prophage composition across clusters and host-associated groups.**

**Figure S20. Distribution of recurrent prophage clusters across sequence types.** Heatmap of clusters present in ≥2 genomes across sequence types. Rows represent clusters and columns represent STs. Distribution patterns indicate both shared and lineage-restricted prophage elements across the multiplex population.

**Figure S21. Genome-wide distribution of prophage clusters across Xylella fastidiosa subsp. multiplex.** Presence–absence heatmap of all prophage clusters (C1–C124), including singleton clusters, across analyzed strains. Rows represent prophage clusters ordered by decreasing cluster size and columns represent individual strains. Presence is shown in blue and absence in orange. Strains are annotated by sequence type and host association. The distribution reveals extensive rarity and heterogeneity in prophage content, with many clusters restricted to individual genomes and a smaller subset shared across multiple lineages.

**Figure S22. Comparative genome architecture of representative prophage clusters.** (A) Conserved architectures among dominant clusters. (B) Plum-associated clusters. (C) Non-plum-associated clusters. Arrows indicate coding sequences and shaded links denote sequence similarity. Conserved and divergent structural patterns are observed across categories.
