# Supplementary material for: Accessory genome dynamics shape lineage-associated diversification in plum-associated Xylella fastidiosa subsp. multiplex
Source: ISME Commun. 2026 Oct 13;6(1):ycag291. doi: 10.1093/ismeco/ycag291 (PMC13755354; doi:10.1093/ismeco/ycag291)
Supplement: Supplementary file 1 [file supplementary_materials_ycag291.zip › Supplementary_Materials_ycag291/Supplementary_info_draft_xfm-plum_ISMEC_revision_FINAL_ycag291.docx]

**Accessory genome dynamics shape the diversification of plum-associated *Xylella fastidiosa* within the global subsp. *multiplex* population**

**SUPPLEMENTARY INFORMATION**

**SUPPLEMENTAL FIGURE LEGENDS**

**Figure S1. Geographic distribution and pathogenicity of plum-associated *Xylella fastidiosa* subsp. *multiplex* isolates.** (A) Geographic origin of isolates included in the study, showing isolates collected from ornamental plum in Riverside, California, and agricultural plum from multiple locations in Texas (Mexia, Overton, and Houston). Individual isolate identifiers are indicated at their respective collection locations. (B) Estimated differences in area under the disease progress curve (AUDPC) for each bacterial isolate relative to the PBS control. Points represent estimated mean differences and horizontal bars represent Dunnett-adjusted 95% confidence intervals. The dashed vertical line at zero indicates no difference from PBS. All bacterial isolates produced significantly greater AUDPC than PBS (Dunnett-adjusted *P* < 0.0001). Isolates are colored according to geographic origin (Texas, red; California, blue).

**Figure S2. Recombination-filtered core-genome phylogeny of *Xylella fastidiosa* subsp. *multiplex*.** Phylogenetic relationships among 49 *X. fastidiosa* subsp. *multiplex* genomes reconstructed after identification and removal of recombinant regions from the core-genome alignment. Sequence types (STs) are indicated adjacent to isolate names. Red indicates plum-associated isolates sequenced in this study, whereas blue indicates publicly available plum-associated isolates included for comparative analysis. The scale bar represents nucleotide substitutions per site.

**Figure S3. Pangenome dynamics and core-genome alignment characteristics of *Xylella fastidiosa* subsp. *multiplex*.** **(A)** Accumulation of pangenome, accessory, core, shell, and cloud gene families across the 49-genome dataset. Curves show changes in gene-family number as genomes were sequentially added across random permutations, with error bars indicating variation among permutations. Continued expansion of the total pangenome and accessory gene fractions with increasing genome number is consistent with an open pangenome. **(B)** Composition of the concatenated core-genome alignment used for phylogenetic reconstruction. The alignment contained 1,545,580 nucleotide sites, including 1,528,960 constant sites (98.92%), 16,620 variable sites (1.08%), and 11,512 parsimony-informative sites (0.74%). Parsimony-informative sites represent a subset of the variable sites.

**Figure S4. Functional category distribution across accessory gene combinations in plum-associated *Xylella fastidiosa* subsp. *multiplex*.** Heatmap showing the proportion (%) of genes assigned to COG functional categories across accessory gene combinations identified in the pangenome analysis. Rows represent individual gene combinations (as defined in the UpSet analysis), and columns correspond to COG categories grouped into major functional classes, including cellular processes and signaling, metabolism, information storage and processing, and poorly characterized functions.

**Figure S5. Functional composition of accessory genomes across lineage groupings in plum-associated *Xylella fastidiosa* subsp. *multiplex*.** Heatmap showing the proportion (%) of genes assigned to COG functional categories across major lineage groupings, including California (CA), Texas (TX), shared (“All”), and unique gene sets. Columns correspond to individual COG categories, grouped into higher-order functional classes (cellular processes and signaling, metabolism, information storage and processing, and poorly characterized functions), and color intensity reflects the relative proportion of each category within a grouping.

**Figure S6. Phylogenetic relationships of pXF51-like and pXF26-like plasmids detected in plum-associated *Xylella fastidiosa* subsp. *multiplex* isolates.** (A) Maximum-likelihood phylogeny of pXF51-like plasmids. (B) Maximum-likelihood phylogeny of pXF26-like plasmids. Trees were reconstructed from plasmid nucleotide alignments using IQ-TREE. Plum isolates sequenced in this study are shown in red, previously reported plum isolates retrieved from NCBI are shown in blue, and the remaining *X. fastidiosa* strains representing other hosts and subspecies are shown in black. Sequence type (ST), host plant, and geographic origin are indicated next to each strain. Both plasmid families were detected among plum-associated *X. fastidiosa* subsp. *multiplex* isolates belonging predominantly to ST10 and ST26.

**Figure S7. Maximum-likelihood phylogeny of pXFAS01-like plasmids.** The tree was reconstructed from plasmid nucleotide alignments using IQ-TREE. Plum isolates sequenced in this study are shown in red, previously reported plum isolates retrieved from NCBI are shown in blue, and the remaining *X. fastidiosa* strains are shown in black. Sequence type (ST), host plant, and geographic origin are indicated next to each strain. Among the plum-associated isolates, pXFAS01-like plasmids were associated predominantly with the ST34 lineage, including California isolates Riv5, OPR27, and OPR29 and Texas isolates Tx-B and Tx-9.

**Figure S8. Phylogenetic relationships of pXF1.3-like plasmids across *Xylella fastidiosa*.** Maximum-likelihood phylogeny of pXF1.3-like plasmid nucleotide sequences. Homologous plasmids were identified based on sequence similarity to the plasmid detected in the plum-associated isolate CFBP8070 and were restricted to small plasmids of comparable size (~1.2–1.3 kb). The CFBP8070 plasmid is highlighted in blue. The phylogeny shows that pXF1.3-like plasmids occur across diverse *X. fastidiosa* subspecies, hosts, geographic origins, and sequence types.

**Figure S9. Pan-plasmid gene presence–absence across the *Xylella fastidiosa* species complex.** Heatmap showing the distribution of orthologous gene clusters identified from pangenome analysis of plasmids across the *X. fastidiosa* species complex. Rows represent individual plasmid sequences and columns represent orthologous gene clusters. Presence (light orange) and absence (navy blue) are indicated for each gene cluster. Hierarchical clustering based on gene content is shown for both plasmids and gene clusters. Row annotations indicate subspecies, host of isolation (plum versus other hosts), and multilocus sequence type (MLST). Plasmids form discrete gene-content clusters broadly corresponding to the sequence-supported plasmid families, while variable gene blocks reflect differences in accessory content among plasmids.

**Figure S10. Comparative gene synteny of pXFAS01-like and pXF64-HB-like plasmids.** Comparative gene synteny among 35 pXFAS01-like plasmids (A) and 2 pXF64-HB-like plasmids (B). pXFAS01-like plasmids show a largely conserved backbone with localized variation in accessory regions, whereas pXF64-HB-like plasmids exhibit extensive collinearity across their length. Arrows represent predicted coding sequences, and shaded links indicate homologous regions and their degree of sequence similarity.

**Figure S11. GC content variation among sequence-supported plasmid families.** GC content of plasmids grouped into pXF1.3-like, pXF26-like, pXFAS01-like, pXF51-like, and pXF64-HB-like families. Individual plasmids are shown within each family, illustrating variation in GC composition within and among plasmid families.

**Figure S12. Functional composition of pXF26-like plasmids.** Proportional distribution of predicted gene functions across pXF26-like plasmids. Mobility-related genes constitute a major functional component, with variable contributions from replication, defense, metabolism, and other functional categories. A substantial fraction of predicted genes remains functionally uncharacterized.

**Figure S13. Functional composition of pXFAS01-like plasmids.** Proportional distribution of predicted gene functions across pXFAS01-like plasmids. Functional profiles show substantial conservation, with mobility-related genes representing a major component and additional contributions from other functional categories. A substantial fraction of predicted genes remains functionally uncharacterized.

**Figure S14. Functional composition of pXF51-like plasmids.** Proportional distribution of predicted gene functions across pXF51-like plasmids. Mobility-related genes are prevalent, together with replication, defense, and other functional components. Variation among plasmids reflects differences in accessory gene content, while a substantial fraction of predicted genes remains functionally uncharacterized.

**Figure S15. Functional composition of pXF64-HB-like plasmids.** Proportional distribution of predicted gene functions across pXF64-HB-like plasmids. Functional profiles are highly similar between plasmids, consistent with the strong conservation of gene content and overall organization observed within this family.

**Figure S16. Distribution of CAZyme families across *Xylella fastidiosa* subsp. *multiplex*.** Presence–absence heatmap of CAZyme families across genomes. Most families are broadly conserved, whereas GH2, GH25, and GH6 show variable presence across the dataset.

**Figure S17. Phylogeny of GH24 proteins across *Xylella fastidiosa* subsp. *multiplex*.** Maximum-likelihood phylogeny of GH24 protein sequences. Proteins from isolates sequenced in this study are shown in red and those from publicly available genomes in blue. Clustering patterns illustrate sequence variation among GH24 homologs and their distribution across different genomic lineages.

**Figure S18. Phylogenetic relationships of GH24 genes across plum-associated *Xylella fastidiosa* subsp. *multiplex* strains.** Circular phylogenetic tree of GH24 gene sequences across plum-associated *X. fastidiosa* subsp. *multiplex* strains. Branch tips are colored by strain identity, and background shading denotes Texas and California plum-associated groups and isolates from other hosts. The tree illustrates sequence variation among GH24 copies and their distribution across different genomic backgrounds.

**Figure S19. Distribution and size characteristics of prophage clusters across *X. fastidiosa* genomes.** (A) Prophage cluster size distribution, showing the number of prophages within each cluster (C1–C124), ranked from largest to smallest. Bars are colored by category: plum-specific (red), shared (gray), and non-plum-specific (blue). (B) Mean prophage length for each cluster, ordered consistently with panel (A). Substantial variation is observed in both cluster size and prophage length, reflecting heterogeneity in prophage composition across clusters and host-associated groups.

**Figure S20. Distribution of recurrent prophage clusters across sequence types.** Heatmap of clusters present in ≥2 genomes across sequence types. Rows represent clusters and columns represent STs. Distribution patterns indicate both shared and lineage-restricted prophage elements across the multiplex population.

**Figure S21. Genome-wide distribution of prophage clusters across Xylella fastidiosa subsp. multiplex.** Presence–absence heatmap of all prophage clusters (C1–C124), including singleton clusters, across analyzed strains. Rows represent prophage clusters ordered by decreasing cluster size and columns represent individual strains. Presence is shown in blue and absence in orange. Strains are annotated by sequence type and host association. The distribution reveals extensive rarity and heterogeneity in prophage content, with many clusters restricted to individual genomes and a smaller subset shared across multiple lineages.

**Figure S22. Comparative genome architecture of representative prophage clusters.** (A) Conserved architectures among dominant clusters. (B) Plum-associated clusters. (C) Non-plum-associated clusters. Arrows indicate coding sequences and shaded links denote sequence similarity. Conserved and divergent structural patterns are observed across categories.

**SUPPLEMENTAL TABLE LEGENDS**

**Table S1. Genome metadata and assembly quality metrics for *Xylella fastidiosa* subsp. *multiplex* strains included in this study.** Summary of strain origin, host, sequence type, genome source, and assembly statistics for genomes used in comparative analyses.

**Table S2. Sensitivity analysis of pangenome composition across Roary protein identity thresholds.** Pangenome composition was compared across 80%, 90%, and 100% protein identity thresholds using the same 49 *Xylella fastidiosa* subsp. *multiplex* genomes. The 80% threshold represents the primary analysis.

**Table S3. Lineage-associated candidate genes distinguishing major plum-associated sequence types.** Candidate genes showing differential presence among the major plum-associated sequence types identified in the comparative genomic analysis.

**Table S4. Plasmid sequences and metadata used in phylogenetic analyses.** Plasmid sequences included in phylogenetic reconstruction, with their plasmid family, plasmid identifier, corresponding *Xylella fastidiosa* strain, sequence type (ST), host, geographic origin, subspecies, GenBank accession, assembled sequence length, and GC content. Plasmids were classified into the pXF1.3-like, pXF26-like, pXFAS01-like, pXF51-like, and pXF64-HB-like families based on the revised plasmid grouping used in this study. Reported plasmid sizes correspond to the assembled sequence lengths used in downstream phylogenetic analyses.

**Table S5. Distribution of predicted conjugation- and type IV secretion-associated genes among major plasmid groups identified in *Xylella fastidiosa*.** Summary of *tra*, *virB/virD*, and other mobility-associated genes detected across the major plasmid families.

**Table S6. BLASTn similarity of representative *Xylella fastidiosa* plasmid families to plasmids from non-*Xylella* bacterial taxa.** Representative plasmids were compared against publicly available sequences to identify related plasmid regions in other bacterial genera.

**Table S7. Distribution of prophage cluster categories across *Xylella fastidiosa* subsp. *multiplex* genomes.** Prophage clusters (C1–C124) categorized based on host distribution as plum-specific, non-plum-specific, or shared. For each category, the number of clusters and cumulative number of prophage sequences are reported, providing an overview of prophage distribution across host-associated lineages.

**Table S8. Characteristics of prophage clusters across *Xylella fastidiosa* genomes.** Prophage sequences grouped into clusters based on sequence similarity and ranked by cluster size (C1–C124). For each cluster, size, host representation (plum vs non-plum), and distribution category are reported. This table highlights variation in cluster prevalence and host association across the dataset.

**Table S9. Distribution and variability of CAZyme families across *Xylella fastidiosa* subsp. *multiplex* genomes.** Detected carbohydrate-active enzyme (CAZyme) families classified by distribution patterns (conserved, variable presence, broadly absent, or variable copy number). Presence rate indicates the proportion of genomes encoding each family, and copy variation reflects variability in gene copy number. CAZyme families are grouped by functional class.

**Table S10. Within-isolate sequence variation among GH24 protein copies in plum-associated *Xylella fastidiosa* subsp. *multiplex* isolates.** GH24 copies were compared at the predicted protein-sequence level. “Unique protein variants” indicates the number of distinct amino acid sequences identified within each isolate. “Identical copies present” indicates whether two or more GH24 copies within an isolate had identical predicted protein sequences. Across all 15 isolates, 105 GH24 copies represented 35 unique protein sequences; 13 identical protein variants were shared between two or more isolates.

**Table S11. Sequence type distribution within prophage clusters across *Xylella fastidiosa* subsp. *multiplex*.** Prophage clusters (C1–C124) annotated with cluster size, host distribution, and associated sequence types (STs). For each cluster, the number of plum and non-plum isolates, ST composition, dominant ST, and distribution pattern (broad, limited, or restricted) are reported, providing insight into lineage-associated patterns of prophage distribution across the *Xfm* dataset.
