# Supplementary material for: Accessory genome dynamics shape lineage-associated diversification in plum-associated Xylella fastidiosa subsp. multiplex
Source: ISME Commun. 2026 Oct 13;6(1):ycag291. doi: 10.1093/ismeco/ycag291 (PMC13755354; doi:10.1093/ismeco/ycag291)
Supplement: Supplementary file 1 [file supplementary_materials_ycag291.zip › Supplementary_Materials_ycag291/Supplementary_tables_xfm-plum_ISMEC_revision1_FINAL_ycag291.docx]

**Table S1. Genome metadata and assembly quality metrics for *Xylella fastidiosa* subsp. *multiplex* strains included in this study.** Summary of strain origin, host, sequence type, genome source, and assembly statistics for genomes used in comparative analyses.

| **Strain** | **Sequence Type** | **Host** | **Isolation Origin** | **^1^ NCBI sequence accession** | **Subspecies** | **BioProject Accession** | **Collection date** | **Total Length (bp)** | **# Contigs** | **N50 (bp)** | **% GC** | **CheckM completeness** | **CheckM contamination** |
| --- | --- | --- | --- | --- | --- | --- | --- | --- | --- | --- | --- | --- | --- |
| AlmaEM3 | ST42 | *Vaccinium* | USA: Georgia | GCF_018069645.1 | *multiplex* | PRJNA720706 | 2011 | 2487451 | 1 | 2487451 | 52 | 100 | 0 |
| AlmaReb6 | ST27 | *Vaccinium* | USA: Georgia | GCA_028621915.1 | *multiplex* | PRJNA930171 | 2019 | 2530311 | 1 | 2530311 | 52 | 95.68 | 0 |
| BB08-1 | ST43 | *Vaccinium* | USA: Florida | GCF_018069665.1 | *multiplex* | PRJNA720706 | 2008 | 2553631 | 1 | 2553631 | 52 | 100 | 0 |
| CFBP13679 | ST10 | *Prunus sp.* | USA: Georgia | GCF_028640385.1 | *multiplex* | PRJNA779852 | 2004 | 2605599 | 316 | 91004 | 51.5 | 100 | 0.21 |
| CFBP8068 | ST41 | *Ulmus sp.* | USA: Washington | GCF_042241895.1 | *multiplex* | PRJNA1026562 | Not reported | 2548602 | 1 | 2548602 | 52 | 100 | 0.01 |
| CFBP8070 | ST10 | *Prunus sp.* | USA: Georgia | GCF_042243345.1 | *multiplex* | PRJNA1026562 | 2004 | 2867959 | 4 | 2796854 | 52 | 100 | 0.24 |
| CFBP8075 | ST27 | *Prunus sp.* | USA: California | GCF_042242545.1 | *multiplex* | PRJNA1026562 | Not reported | 2439524 | 1 | 2439524 | 52 | 100 | 0 |
| CFBP8173 | ST41 | *Prunus salicina* | USA: Georgia | GCF_042241145.1 | *multiplex* | PRJNA1026562 | Not reported | 2548622 | 1 | 2548622 | 52 | 100 | 0.01 |
| CFBP8416 | ST7 | *Polygala myrtifolia* | France: Propriano | GCF_001971475.1 | *multiplex* | PRJNA314983 | 2015 | 2506765 | 1 | 2506765 | 52 | 100 | 0 |
| CFBP8417 | ST6 | *Spartium junceum* | France: Alata | GCF_001971505.2 | *multiplex* | PRJNA314984 | 2015 | 2672242 | 1 | 2672242 | 52 | 100 | 0.03 |
| CFBP8418 | ST6 | *Spartium junceum* | France: Alata | GCF_001971465.2 | *multiplex* | PRJNA314986 | 2015 | 2797446 | 4 | 1679482 | 52 | 100 | 0.04 |
| CFBP8430 | ST6 | *Polygala myrtifolia* | France | GCF_028498295.1 | *multiplex* | PRJNA780364 | 2015 | 2845597 | 4 | 2716895 | 52 | 100 | 1.14 |
| CFBP8431 | ST6 | *Prunus cerasifera* | France | GCF_028498215.1 | *multiplex* | PRJNA780364 | 2015 | 2726288 | 2 | 1987931 | 52 | 100 | 2 |
| CFBP8432 | ST7 | *Polygala myrtifolia* | France | GCF_028752755.1 | *multiplex* | PRJNA780364 | 2015 | 2560753 | 1 | 2560653 | 52 | 100 | 0.04 |
| CFBP8433 | ST7 | *Cistus monspeliensis* | France | GCF_028752655.1 | *multiplex* | PRJNA780364 | 2015 | 2496778 | 1 | 2496778 | 52 | 100 | 0 |
| CFBP8435 | ST7 | *Coronilla valentina* | France | GCF_028498235.1 | *multiplex* | PRJNA780364 | 2015 | 2765940 | 3 | 2672324 | 52 | 100 | 0.03 |
| Dixon | ST6 | *Prunus dulcis* | USA: California | GCF_029626005.1 | *multiplex* | PRJNA949737 | Not reported | 2725944 | 9 | 671290 | 52 | 100 | 0.12 |
| ESVL | ST6 | *Prunus dulcis* | Spain: Alicante | GCF_004023385.2 | *multiplex* | PRJNA482360 | 2017-08-08 | 2613641 | 3 | 2552692 | 52 | 100 | 0.01 |
| Fillmore | ST81 | *Olea europaea* | USA: California | GCF_012974105.1 | *multiplex* | PRJNA627683 | 2009-08 | 2526544 | 1 | 2526544 | 52 | 100 | 0.01 |
| IAS-AXF64H11 | ST6 | *Not reported* | Spain: Alicante | GCF_046605575.1 | *multiplex* | PRJNA1199929 | 2018 | 2712445 | 1 | 2712445 | 52.5 | 100 | 0 |
| IVIA5901 | ST6 | *Prunus dulcis* | Spain: Alicante | GCF_004023395.2 | *multiplex* | PRJNA482385 | 2017-09-20 | 2559157 | 1 | 2559157 | 52 | 100 | 0.16 |
| LA-Y3C | ST43 | *Vaccinium virgatum* | USA: Louisiana | GCF_021459845.1 | *multiplex* | PRJNA672949 | 2013 | 2526390 | 1 | 2526390 | 52 | 100 | 0 |
| LM10 | ST7 | *Olea europaea* | USA: California | GCF_012974145.1 | *multiplex* | PRJNA627683 | 2011-05 | 2669650 | 1 | 2669650 | 52 | 100 | 0.01 |
| M12 | ST7 | *Olea europaea* | USA: California | GCF_012974105.1 | *multiplex* | PRJNA627683 | 2009-08 | 2526544 | 1 | 2526544 | 52 | 100 | 0.01 |
| NZ13 | ST6 | *Not reported* | USA: California | GCF_043195255.1 | *multiplex* | PRJNA1086547 | 2020-10-17 | 2673509 | 5 | 2559549 | 51.5 | 100 | 0.12 |
| NZ4 | ST88 | *Not reported* | USA: California | GCF_043355825.1 | *multiplex* | PRJNA1084903 | 2020-10-17 | 2610277 | 3 | 2586758 | 52 | 100 | 0.19 |
| Oak_35874 | ST9 | *Quercus* | USA: Washington DC | GCF_021459905.1 | *multiplex* | PRJNA672949 | Not reported | 2648027 | 2 | 2621666 | 52 | 100 | 0.01 |
| OPR27 | ST34 | *Prunus cerasifera* | USA: California | SRR37938477; SRR37938469 | *multiplex* | PRJNA1449158 | 2007 | 2538831 | 1 | 2538831 | 53 | 100 | 0 |
| OPR29 | ST34 | *Prunus cerasifera* | USA: California | SRR37938476; SRR37938468 | *multiplex* | PRJNA1449158 | 2007 | 2558720 | 1 | 2558720 | 54 | 100 | 0 |
| RAAR14 | ST26 | *Prunus domestica* | Brazil: Veranopolis | GCF_009695495.1 | *multiplex* | PRJNA545878 | 2010 | 2543559 | 192 | 96594 | 51.5 | 100 | 0.08 |
| Red_Oak_2 | ST7 | *Quercus rubra* | USA: Georgia | GCF_015475935.1 | *multiplex* | PRJNA672949 | 2006 | 2476446 | 1 | 2476446 | 52 | 100 | 0.01 |
| Red_Oak_8 | ST7 | *Quercus rubra* | USA: Georgia | GCF_021459885.1 | *multiplex* | PRJNA672949 | 2006 | 2476438 | 1 | 2476438 | 52 | 100 | 0.01 |
| RH1 | ST7 | *Olea europaea* | USA: California | GCF_012974125.1 | *multiplex* | PRJNA627683 | 2008-10 | 2678425 | 1 | 2678425 | 52 | 100 | 0.28 |
| RIV5 | ST34 | *Prunus cerasifera* | USA: California | GCF_015475955.1 | *multiplex* | PRJNA672949 | 2006 | 2538293 | 2 | 2499994 | 52 | 100 | 0 |
| Santa29b | ST81 | *Santolina chamaecyparissus* | Spain: Menorca | GCF_042240315.1 | *multiplex* | PRJNA1026562 | 2022 | 2593199 | 1 | 2593199 | 52 | 100 | 0.01 |
| TX-2 | ST26 | *Prunus salicina* | USA: Texas | SRR37938485; SRR37938487 | *multiplex* | PRJNA1449158 | 2022 | 2671761 | 6 | 2087522 | 52 | 100 | 0.06 |
| TX-467 | ST26 | *Prunus salicina* | USA: Texas | SRR37938482; SRR37938474 | *multiplex* | PRJNA1449158 | 2022 | 2837517 | 11 | 490337 | 52 | 100 | 0.34 |
| TX-468 | ST26 | *Prunus salicina* | USA: Texas | SRR37938481; SRR37938473 | *multiplex* | PRJNA1449158 | 2022 | 2710835 | 6 | 2227982 | 52 | 100 | 0.2 |
| TX-470 | ST26 | *Prunus salicina* | USA: Texas | SRR37938472; SRR37938472 | *multiplex* | PRJNA1449158 | 2022 | 2851165 | 9 | 1045974 | 52 | 100 | 0.34 |
| TX-471 | ST26 | *Prunus salicina* | USA: Texas | SRR37938479; SRR37938471 | *multiplex* | PRJNA1449158 | 2022 | 2800560 | 5 | 2163497 | 52 | 100 | 0.2 |
| TX-9 | ST34 | *Prunus salicina* | USA: Texas | SRR37938484; SRR37938486 | *multiplex* | PRJNA1449158 | 2022 | 2581428 | 4 | 2388799 | 52 | 100 | 0 |
| TX-B | ST34 | *Prunus salicina* | USA: Texas | SRR37938475; SRR37938483 | *multiplex* | PRJNA1449158 | 2022 | 2551963 | 2 | 2513665 | 52 | 100 | 0 |
| XF3348 | ST81 | *Prunus dulcis* | Spain: Mallorca | GCF_009669515.1 | *multiplex* | PRJNA544690 | 2018-07 | 2573395 | 392 | 102161 | 52 | 100 | 0.02 |
| XYL1752-17 | ST7 | *Prunus dulcis* | Spain: Menorca | GCF_042239055.1 | *multiplex* | PRJNA1026562 | 2017-07 | 2769601 | 1 | 2769601 | 52 | 100 | 0.84 |
| XYL1966-18 | ST7 | *Olea europaea* | Spain: Menorca | GCF_014856865.1 | *multiplex* | PRJNA605223 | 2018-08 | 2514978 | 125 | 78976 | 52 | 100 | 0.24 |
| XYL1968-18 | ST81 | *Olea europaea var. europaea* | Spain: Menorca | GCF_048564875.1 | *multiplex* | PRJNA1026562 | 2018-08 | 2,673,021 | 1 | 2673021 | 52 | 100 | 0.01 |
| XYL1981 | ST7 | *Ficus carica* | Spain: Mallorca | GCF_009669455.1 | *multiplex* | PRJNA544690 | 2017-08 | 2554510 | 353 | 96968 | 52 | 100 | 0.53 |
| XYL466-19 | ST81 | *Olea europaea var. sylvestris* | Spain: Mallorca | GCF_042237735.1 | *multiplex* | PRJNA1026562 | 2019 | 2790648 | 2 | 1914251 | 52.5 | 100 | 0.03 |
| XYL468 | ST81 | *Olea europaea var. sylvestris* | Spain: Mallorca | GCF_042237065.1 | *multiplex* | PRJNA1026562 | 2019 | 2809670 | 2 | 1906161 | 52 | 100 | 0.04 |

**^1^**For publicly available genomes, GCA/GCF identifiers represent GenBank or RefSeq assembly accessions. For isolates sequenced in this study, the two SRR identifiers represent Nanopore and Illumina raw reads, respectively, deposited in the NCBI SRA. Draft genomes were assembled locally from these reads but were not deposited as GenBank assemblies; therefore, assembly statistics and CheckM2 estimates refer to the locally generated assemblies.

**Table S2. Sensitivity analysis of pangenome composition across Roary protein identity thresholds.** Pangenome composition was compared across 80%, 90%, and 100% protein identity thresholds using the same 49 *Xylella fastidiosa* subsp. *multiplex* genomes. The 80% threshold represents the primary analysis.

| **Identity threshold** | **Core** | **Soft core** | **Shell** | **Cloud** | **Total genes** |
| --- | --- | --- | --- | --- | --- |
| **80% (primary analysis)** | 1,606 | 207 | 1,340 | 3,570 | 6,723 |
| **90%** | 1,587 | 227 | 1,362 | 3,682 | 6,858 |
| **100%** | 987 | 483 | 1,609 | 4,783 | 7,862 |

**Table S3. Lineage-associated candidate genes distinguishing major plum-associated sequence types.** Candidate genes showing differential presence among the major plum-associated sequence types identified in the comparative genomic analysis.

| **Lineage (ST)** | **Gene** | **Annotation** | **Genomic context** | **Functional category** | **Observed pattern** | **Biological relevance** |
| --- | --- | --- | --- | --- | --- | --- |
| ST26 (TX-rich) | *zot* | Zonula occludens toxin | Prophage-associated | Host interaction / virulence | Present in ST26 accessory genome | May enhance host interaction and contribute to increased virulence |
|  | *xerD* | Integrase/recombinase | Prophage-associated | Genome plasticity | Enriched in ST26 accessory genome | Supports prophage integration and genome rearrangement |
|  | Baseplate J-like proteins | Phage structural proteins | Prophage-associated | Phage-host interaction | Lineage-associated | May influence phage adsorption and host specificity |
|  | *nicK* | DNA relaxase | Plasmid-associated | Conjugation / mobility | Present in ST26 lineage | Indicates enhanced horizontal gene transfer capacity |
|  | XRE regulator | Transcriptional regulator | Chromosomal/mobile | Regulatory | Shared in ST26 lineage | May alter transcriptional responses and stress adaptation |
|  | VapC | Toxin RNase | Chromosomal | Stress response | Lineage-associated | May contribute to reduced growth and persistence under stress |
|  | *yfdQ* | Stress-associated protein | Chromosomal | Stress response | Accessory-enriched | May regulate replication under stress |
| ST26-specific | *higA* | Antitoxin | Chromosomal | Regulatory | Subgroup-specific | Linked to toxin–antitoxin regulation of virulence |
| ST34 (CA-rich) | RTX toxin | Pore-forming toxin | Chromosomal | Host interaction | Present in ST34 lineage | Involved in host colonization and virulence |
|  | Lipid A acyltransferase | Envelope biosynthesis enzyme | Chromosomal | Cell envelope | Lineage-associated | May influence membrane structure and biofilm anchoring |
|  | *bglX* | Beta-glucosidase | Chromosomal | Carbohydrate metabolism | Shared in ST34 lineage | Enables sugar utilization from host tissues |
|  | *gloB* | Metallo-hydrolase | Chromosomal | Detoxification | Shared in ST34 lineage | Supports metabolic homeostasis |

**Table S4. Plasmid sequences and metadata used in phylogenetic analyses.** Plasmid sequences included in phylogenetic reconstruction, with their plasmid family, plasmid identifier, corresponding *Xylella fastidiosa* strain, sequence type (ST), host, geographic origin, subspecies, GenBank accession, assembled sequence length, and GC content. Plasmids were classified into the pXF1.3-like, pXF26-like, pXFAS01-like, pXF51-like, and pXF64-HB-like families based on the revised plasmid grouping used in this study. Reported plasmid sizes correspond to the assembled sequence lengths used in downstream phylogenetic analyses.

| **Plasmid family** | **Plasmid name** | **Strain** | **Sequence Type** | **Host** | **Origin** | **Subspecies** | **Accession** | **Length (bp)** | **GC (%)** |
| --- | --- | --- | --- | --- | --- | --- | --- | --- | --- |
| pXF1.3-like | pUCLA ESVL | ESVL | ST6 | Almond | Spain | *multiplex* | CP099518.1 | 1289 | 54.69 |
| pXF1.3-like | pXF pUCLA | UCLA | ST1 | Almond | Spain | *multiplex* | CP099518.1 | 1289 | 54.69 |
| pXF1.3-like | pXF pUCLAa | UCLA | ST31 | Mulberry | California, USA | *morus* | DQ063224.1 | 1298 | 54.62 |
| pXF1.3-like | pXF pUCLAb | UCLA | ST1 | Grapevine | California, USA | *fastidiosa* | DQ063225.1 | 1293 | 54.76 |
| pXF1.3-like | pXF pUCLAc | UCLA | ST1 | Grapevine | California, USA | *fastidiosa* | DQ063226.1 | 1284 | 54.52 |
| pXF1.3-like | pXF-P3 CFBP8070 | CFBP8070 | ST10 | Plum | Georgia, USA | *multiplex* | CP136978.1 | 1286 | 54.59 |
| pXF1.3-like | pXF-p2-BA GR31 | GR31 | ST1 | Grapevine | Italy | *fastidiosa* | CP172099.1 | 1286 | 54.09 |
| pXF1.3-like | pXF1.3 9a5c | 9a5c | ST13 | Citrus | Brazil | *pauca* | AE003850.3 | 1286 | 55.56 |
| pXF1.3-like | pXF1.3 Riv13 | Riv13 | ST1 | Redbud | California, USA | *fastidiosa* | CP090517.1 | 1380 | 53.48 |
| pXF1.3-like | pXF1.3 Riv16 | Riv16 | ST1 | Grapevine | California, USA | *fastidiosa* | CP090318.1 | 1318 | 53.79 |
| pXF1.3-like | pXF868 | pXF868 | ST2 | Grapevine | N/Carolina, USA | *fastidiosa* | U71220.1 | 1296 | 54.86 |
| pXF1.3-like | pXFPD1.3 Temecula1 | Temecula1 | ST1 | Grapevine | California, USA | *fastidiosa* | AE009443.1 | 1346 | 53.79 |
| pXF26-like | pCFBP8498 | pCFBP8498 | ST73 | Coffee | Costa Rica | *pauca* | NZ_JBSSSO010000005 | 28518 | 57.08 |
| pXF26-like | pXF GB514 | GB514 | ST1 | Grapevine | Texas, USA | *fastidiosa* | CP002166.1 | 26180 | 49.34 |
| pXF26-like | pXF MUL0034 | MUL0034 | ST30 | Mulberry | California, USA | *morus* | CP006739.1 | 24391 | 49 |
| pXF26-like | pXF RIV11 | RIV11 | ST31 | Mulberry | California, USA | *morus* | GU938457.1 | 25105 | 49.03 |
| pXF26-like | pXF RIV19 | RIV19 | ST62 | Mulberry | California, USA | *morus* | GU938459.1 | 24372 | 49.02 |
| pXF26-like | pXF Riv16 | Riv16 | ST31 | Mulberry | California, USA | *morus* | CP090317.1 | 25102 | 49.02 |
| pXF26-like | pXF Tx2 | Tx-2 | ST26 | Plum | Texas, USA | *multiplex* | This study | 26329 | 49.36 |
| pXF26-like | pXF XYL1981-17 | XYL1981-17 | ST1 | Redbud | California, USA | *fastidiosa* | CP183394.1 | 22595 | 49.61 |
| pXF26-like | pXF-Ann-1 | Ann-1 | ST5 | Oleander | California, USA | *sandyi* | CP006697.1 | 30305 | 49.48 |
| pXF26-like | pXF-P1 RAAR14 | RAAR14 | ST26 | Plum | Brazil | *multiplex* | NZ_VDDF01000167.1 | 26405 | 49.35 |
| pXF26-like | pXF-P1 Tx467 | Tx-467 | ST26 | Plum | Texas, USA | *multiplex* | This study | 28098 | 49.41 |
| pXF26-like | pXF-P1 Tx468 | Tx-468 | ST26 | Plum | Texas, USA | *multiplex* | This study | 26329 | 49.36 |
| pXF26-like | pXF-P1 Tx470 | Tx-470 | ST26 | Plum | Texas, USA | *multiplex* | This study | 28098 | 49.41 |
| pXF26-like | pXF-P1 Tx471 | Tx-471 | ST26 | Plum | Texas, USA | *multiplex* | This study | 28098 | 49.41 |
| pXF26-like | pXF-P1.COF0324 | COF0324 | ST14 | Coffee | Brazil | *pauca* | CM003759.1 | 26508 | 49.1 |
| pXF26-like | pXF-P1.COF0407 | COF0407 | ST53 | Coffee | Costa Rica | *pauca* | CM003743.1 | 26571 | 48.76 |
| pXF26-like | pXF-P1.CVC0251 | CVC0251 | ST12 | Citrus | Brazil | *pauca* | CM003755.1 | 27016 | 49.39 |
| pXF26-like | pXF-P1.OLS0478 | OLS0478 | ST53 | Oleander | Costa Rica | *pauca* | CM003752.1 | 31618 | 49.35 |
| pXF26-like | pXF-P2 CFBP8070 | CFBP8070 | ST10 | Plum | Georgia, USA | *multiplex* | CP136977.1 | 26328 | 49.35 |
| pXF26-like | pXF-P2 CFBP8073 | CFBP8073 | ST2 | Grapevine | N/Carolina, USA | *fastidiosa* | CP109889.1 | 28508 | 49.53 |
| pXF26-like | pXF26 Oak35874 | Oak 35874 | ST9 | Oak | DC, USA | *multiplex* | CP090512.1 | 26361 | 49.41 |
| pXF26-like | pXF27-J1 J1a12 | J1a12 | ST11 | Citrus | Brazil | *pauca* | CP009824.1 | 27268 | 49.42 |
| pXF26-like | pXF27b1-B111 | B111 | ST12 | Citrus | Brazil | *pauca* | NZ_JWJX01000023 | 27256 | 49.42 |
| pXF26-like | pXF30-OC8 | OC8 | ST5 | Olive | California, USA | *sandyi* | CP090548.1 | 30305 | 49.48 |
| pXFAS01-like | p1 BA GR31 | GR31 | ST1 | Grapevine | Italy | *fastidiosa* | CP172098.1 | 38298 | 49.21 |
| pXFAS01-like | pBA AL21 | AL21 | ST1 | Almond | Apulia, Italy | *fastidiosa* | CP172096.1 | 38298 | 49.21 |
| pXFAS01-like | pSal1 Salento-1 | Salento-1 | ST53 | Olive | Apulia, Italy | *pauca* | CP016609.1 | 36269 | 49.58 |
| pXFAS01-like | pSal2 Salento-2 | Salento-2 | ST53 | Olive | Apulia, Italy | *pauca* | CP016611.1 | 36270 | 49.58 |
| pXFAS01-like | pXF CFBP8478 | CFBP8478 | ST53 | Olive | Costa Rica | *pauca* | CP135135.1 | 47863 | 51.42 |
| pXFAS01-like | pXF CO33 | CO33 | ST72 | Coffee | Costa Rica | *pauca* | CP135133.1 | 47863 | 51.43 |
| pXFAS01-like | pXF FB7 | FB7 | ST69 | Citrus | Argentina | *pauca* | CP014330.2 | 39408 | 49.95 |
| pXFAS01-like | pXF ICMP8742 | ICMP8742 | ST2 | Elm | Unknown | *fastidiosa* | CP117824.1 | 41752 | 49.45 |
| pXFAS01-like | pXF Riv5 | Riv5 | ST34 | Plum | California, USA | *multiplex* | CP064327.1 | 38299 | 49.21 |
| pXFAS01-like | pXF-De Donno | De Donno | ST53 | Olive | Apulia, Italy | *pauca* | CP020871.1 | 35273 | 49.58 |
| pXFAS01-like | pXF-OPR27 | OPR27 | ST34 | Plum | California, USA | *multiplex* | This study | 38298 | 49.21 |
| pXFAS01-like | pXF-OPR29 | OPR29 | ST34 | Plum | California, USA | *multiplex* | This study | 38298 | 49.21 |
| pXFAS01-like | pXF-Tx9 | Tx-9 | ST26 | Plum | Texas, USA | *multiplex* | This study | 38296 | 49.21 |
| pXFAS01-like | pXF-TxB | Tx-B | ST26 | Plum | Texas, USA | *multiplex* | This study | 38298 | 49.2 |
| pXFAS01-like | pXF879-41 ATCC35879 | ATCC35879 | ST2 | Grapevine | Florida, USA | *fastidiosa* | CP044353.1 | 41753 | 49.45 |
| pXFAS01-like | pXF879-41 ICMP15197 | ICMP15197 | ST2 | Grapevine | Florida, USA | *fastidiosa* | CP118540.1 | 41752 | 49.45 |
| pXFAS01-like | pXFAS CFBP7970 | CFBP7970 | ST2 | Grapevine | Florida, USA | *fastidiosa* | CP179748.1 | 83542 | 49.47 |
| pXFAS01-like | pXFAS CFBP8082 | CFBP8082 | ST2 | Ragweed | Florida, USA | *fastidiosa* | CP179746.1 | 41752 | 49.45 |
| pXFAS01-like | pXFAS R2XF4358-18 | R2XF4358-18 | ST1 | Buckthorn | Spain | *fastidiosa* | CP109899.1 | 38297 | 49.21 |
| pXFAS01-like | pXFAS XYL2014-18 | XYL2014-18 | ST1 | Almond | Spain | *fastidiosa* | CP179744.1 | 38300 | 49.21 |
| pXFAS01-like | pXFAS XYL2017-18 | XYL2017-18 | ST1 | Almond | Spain | *fastidiosa* | CP179742.1 | 38298 | 49.21 |
| pXFAS01-like | pXFAS XYL2093-18 | XYL2093-18 | ST1 | Almond | Spain | *fastidiosa* | CP179738.1 | 38297 | 49.21 |
| pXFAS01-like | pXFAS XYL2153-18 | XYL2153-18 | ST1 | Grapevine | Spain | *fastidiosa* | CP179740.1 | 38295 | 49.2 |
| pXFAS01-like | pXFAS XYL461 | XYL461 | ST26 | Buckthorn | Spain | *fastidiosa* | CP109896.1 | 38297 | 49.21 |
| pXFAS01-like | pXFAS-5235 IVIA5235 | IVIA5235 | ST1 | Grapevine | Spain | *fastidiosa* | CP047172.1 | 38297 | 49.21 |
| pXFAS01-like | pXFAS-5770 IVIA5770 | IVIA5770 | ST1 | Grapevine | Spain | *fastidiosa* | CP109893.1 | 38297 | 49.21 |
| pXFAS01-like | pXFAS01 Bakersfield-1 | Bakersfield-1 | ST1 | Grapevine | California, USA | *fastidiosa* | CP040800.2 | 38298 | 49.21 |
| pXFAS01-like | pXFAS01 Bakersfield-13 | Bakersfield-13 | ST1 | Grapevine | California, USA | *fastidiosa* | CP064331.1 | 38293 | 49.21 |
| pXFAS01-like | pXFAS01 Bakersfield-8 | Bakersfield-8 | ST1 | Grapevine | California, USA | *fastidiosa* | CP064335.1 | 38292 | 49.21 |
| pXFAS01-like | pXFAS01 CFBP8430 | CFBP8430 | ST6 | Myrtle-leaf | France | *multiplex* | NZ_JAJKGO010000004 | 38772 | 49.1 |
| pXFAS01-like | pXFAS01 CP109895 | CP109895 | ST1 | Grapevine | California, USA | *fastidiosa* | CP109895.1 | 38294 | 49.2 |
| pXFAS01-like | pXFAS01 ICMP8731 | ICMP8731 | ST1 | Grapevine | ATCC, USA | *fastidiosa* | CP117822.1 | 38298 | 49.21 |
| pXFAS01-like | pXFAS01 M23 | M23 | ST16 | Almond | California, USA | *fastidiosa* | CP001012.1 | 38297 | 49.2 |
| pXFAS01-like | pXFAS01 Riv13 | Riv13 | ST1 | Redbud | California, USA | *fastidiosa* | CP090516.1 | 38096 | 49.19 |
| pXFAS01-like | pXFAS01-CFBP8351 | CFBP8351 | ST1 | Grapevine | California, USA | *fastidiosa* | CP109895.1 | 38294 | 49.2 |
| pXFAS01-like | pXFAS01-Dixon | Dixon | ST6 | Almond | California, USA | *multiplex* | NZ_JARTVJ010000009 | 38298 | 49.21 |
| pXF51-like | pXF-P1 CFBP8070 | CFBP8070 | ST10 | Plum | Georgia, USA | *multiplex* | CP136976.1 | 43491 | 48.9 |
| pXF51-like | pXF-P1 CFBP8073 | CFBP8073 | ST10 | Coffee | France | *fastidiosa* | CP109888.1 | 38041 | 49.23 |
| pXF51-like | pXF-P2 RAAR14 | RAAR14 | ST26 | Plum | Brazil | *multiplex* | NZ_VDDF01000173.1 | 41960 | 49.01 |
| pXF51-like | pXF-P2 Tx467 | Tx-467 | ST26 | Plum | Texas, USA | *multiplex* | This study | 43491 | 48.89 |
| pXF51-like | pXF-P2 Tx468 | Tx-468 | ST26 | Plum | Texas, USA | *multiplex* | This study | 43491 | 48.89 |
| pXF51-like | pXF-P2 Tx470 | Tx-470 | ST26 | Plum | Texas, USA | *multiplex* | This study | 43491 | 48.89 |
| pXF51-like | pXF-P2 Tx471 | Tx-471 | ST26 | Plum | Texas, USA | *multiplex* | This study | 43491 | 48.89 |
| pXF51-like | pXF39 Pr8x | Pr8x | ST26 | Plum | Brazil | *multiplex* | CP009827.1 | 39580 | 49.55 |
| pXF51-like | pXF51 9a5c | 9a5c | ST13 | Citrus | Brazil | *pauca* | AE003851.1 | 51158 | 49.57 |
| pXF51-like | pXF51-J1 J1a12 | J1a12 | ST16 | Citrus | Brazil | *pauca* | CP009825.1 | 51180 | 49.58 |
| pXF51-like | pXF51ud U24D | U24D | ST13 | Citrus | Brazil | *pauca* | CP009791.1 | 51156 | 49.57 |
| pXF64-HB-like | P60_ESVL | ESVL | ST6 | Almond | Spain | *multiplex* | CP099517.1 | 59660 | 52.59 |
| pXF64-HB-like | P60_Hib4 | Hib4 | ST70 | Hibiscus | Brazil | *pauca* | CP009886.1 | 64251 | 52.29 |

**Table S5. Distribution of predicted conjugation- and type IV secretion-associated genes among major plasmid groups identified in *Xylella fastidiosa*.** Summary of *tra*, *virB/virD*, and other mobility-associated genes detected across the major plasmid families.

| **Plasmid group** | ***tra* genes** | ***virB*/*virD* genes** | **Other mobility-associated genes** | **Within-group pattern** | **Annotation-based interpretation** |
| --- | --- | --- | --- | --- | --- |
| **pXF26-like** | Variable; including *traG*, *traL* | Variable; including *virB3, virB4, virB5, virB6, virB8, virB9, virB10, virB11, virD2,* and *virD4* | *ptlE* in some plasmids | Heterogeneous | Extensive but variable predicted transfer/T4SS machinery |
| **pXFAS01-like** | *traB, traC, traD, traG, traI, traJ, traK, traL,* and *traM* | *virB4, virB10* | Putative conjugal transfer protein | Highly conserved | Extensive predicted conjugation-associated machinery |
| **pXF51-like** | None consistently detected | *virB1, virB3, virB4, virB5, virB6, virB8, virB9, virB10, virB11,* and *virD4* | — | Highly conserved | Extensive predicted T4SS machinery |
| **pXF64-HB-like** | *traI* | None detected | — | Conserved in the two plasmids examined | Limited transfer-associated machinery detected |

**Table S6. BLASTn similarity of representative *Xylella fastidiosa* plasmid families to plasmids from non-*Xylella* bacterial taxa.** Representative plasmids were compared against publicly available sequences to identify related plasmid regions in other bacterial genera.

| ***Xfm* plasmid family** | **Query representative** | **Non-*Xylella* taxon** | **Subject plasmid** | **Accession** | **Identity (%)** | **Coverage (%)** |
| --- | --- | --- | --- | --- | --- | --- |
| pXF1.3-like | pXF1.3-9a5c | No significant match recovered | — | — | — | — |
| pXF26-like | pXF26-Oak35874 | *Verminephrobacter eiseniae* | pVEIS01 | CP000543.1 | 78.19–84.77 | 62 |
| pXFAS01-like | pXFAS01-CFBP8430 | Uncultured bacterium KIFA123 | pKIFA123 | LC852818.1 | 76.28–80.26 | 58 |
| pXFAS01-like | pXFAS01-CFBP8430 | *Xanthomonas phaseoli* pv. *manihotis* | pXAM48 | CP083579.1 | 75.39–78.81 | 43 |
| pXF51-like | pXF51-9a5c | Uncultured bacterium KIFA123 | pKIFA123 | LC852818.1 | 80.44 | 16 |
| pXF51-like | pXF51-9a5c | *Xanthomonas phaseoli* pv. *manihotis* | pXAM48 | CP083579.1 | 77.43–81.30 | 6 |
| pXF64-HB-like | pXF64-HB-Hib4 | *Paraburkholderia* sp. A1BS-2L | pTA2.2 | CP120433.1 | 81.59–87.98 | 66 |
| pXF64-HB-like | pXF64-HB-Hib4 | *Paraburkholderia hospita* | pEMT1 | CP026110.1 | 81.59–87.98 | 66 |

**Table S7. Distribution of prophage cluster categories across *Xylella fastidiosa* subsp. *multiplex* genomes.** Prophage clusters (C1–C124) categorized based on host distribution as plum-specific, non-plum-specific, or shared. For each category, the number of clusters and cumulative number of prophage sequences are reported, providing an overview of prophage distribution across host-associated lineages.

| **Category** | **Number of clusters** | **Cumulative prophage sequences** |
| --- | --- | --- |
| plum-specific | 16 | 57 |
| non-plum-specific | 82 | 204 |
| shared | 26 | 253 |

**Table S8. Characteristics of prophage clusters across *Xylella fastidiosa* genomes.** Prophage sequences grouped into clusters based on sequence similarity and ranked by cluster size (C1–C124). For each cluster, size, host representation (plum vs non-plum), and distribution category are reported. This table highlights variation in cluster prevalence and host association across the dataset.

| **Cluster ID** | **Original cluster** | **Cluster size** | **Num plum** | **Num nonplum** | **Category** |
| --- | --- | --- | --- | --- | --- |
| C1 | Cluster_113 | 39 | 10 | 29 | shared |
| C2 | Cluster_46 | 36 | 6 | 31 | shared |
| C3 | Cluster_87 | 34 | 8 | 26 | shared |
| C4 | Cluster_94 | 28 | 4 | 24 | shared |
| C5 | Cluster_121 | 14 | 0 | 12 | non-plum-specific |
| C6 | Cluster_91 | 13 | 8 | 1 | shared |
| C7 | Cluster_93 | 11 | 5 | 0 | plum-specific |
| C8 | Cluster_30 | 10 | 1 | 10 | shared |
| C9 | Cluster_117 | 9 | 0 | 6 | non-plum-specific |
| C10 | Cluster_100 | 7 | 4 | 3 | shared |
| C11 | Cluster_104 | 7 | 0 | 6 | non-plum-specific |
| C12 | Cluster_108 | 7 | 0 | 7 | non-plum-specific |
| C13 | Cluster_114 | 7 | 0 | 7 | non-plum-specific |
| C14 | Cluster_118 | 7 | 0 | 7 | non-plum-specific |
| C15 | Cluster_29 | 7 | 0 | 5 | non-plum-specific |
| C16 | Cluster_40 | 7 | 3 | 5 | shared |
| C17 | Cluster_86 | 7 | 5 | 1 | shared |
| C18 | Cluster_101 | 6 | 6 | 0 | plum-specific |
| C19 | Cluster_122 | 6 | 0 | 5 | non-plum-specific |
| C20 | Cluster_28 | 6 | 1 | 6 | shared |
| C21 | Cluster_32 | 6 | 0 | 6 | non-plum-specific |
| C22 | Cluster_37 | 6 | 1 | 6 | shared |
| C23 | Cluster_39 | 6 | 1 | 5 | shared |
| C24 | Cluster_6 | 6 | 4 | 2 | shared |
| C25 | Cluster_60 | 6 | 1 | 6 | shared |
| C26 | Cluster_61 | 6 | 0 | 3 | non-plum-specific |
| C27 | Cluster_66 | 6 | 1 | 5 | shared |
| C28 | Cluster_96 | 6 | 4 | 0 | plum-specific |
| C29 | Cluster_105 | 5 | 3 | 2 | shared |
| C30 | Cluster_48 | 5 | 0 | 1 | non-plum-specific |
| C31 | Cluster_85 | 5 | 5 | 0 | plum-specific |
| C32 | Cluster_88 | 5 | 0 | 4 | non-plum-specific |
| C33 | Cluster_98 | 5 | 5 | 0 | plum-specific |
| C34 | Cluster_124 | 4 | 0 | 4 | non-plum-specific |
| C35 | Cluster_17 | 4 | 3 | 1 | shared |
| C36 | Cluster_26 | 4 | 2 | 2 | shared |
| C37 | Cluster_27 | 4 | 0 | 4 | non-plum-specific |
| C38 | Cluster_31 | 4 | 0 | 4 | non-plum-specific |
| C39 | Cluster_36 | 4 | 0 | 4 | non-plum-specific |
| C40 | Cluster_41 | 4 | 0 | 3 | non-plum-specific |
| C41 | Cluster_45 | 4 | 3 | 1 | shared |
| C42 | Cluster_59 | 4 | 1 | 4 | shared |
| C43 | Cluster_64 | 4 | 0 | 4 | non-plum-specific |
| C44 | Cluster_74 | 4 | 3 | 1 | shared |
| C45 | Cluster_90 | 4 | 3 | 0 | plum-specific |
| C46 | Cluster_99 | 4 | 3 | 0 | plum-specific |
| C47 | Cluster_107 | 3 | 1 | 2 | shared |
| C48 | Cluster_18 | 3 | 3 | 0 | plum-specific |
| C49 | Cluster_35 | 3 | 0 | 3 | non-plum-specific |
| C50 | Cluster_49 | 3 | 0 | 3 | non-plum-specific |
| C51 | Cluster_50 | 3 | 0 | 3 | non-plum-specific |
| C52 | Cluster_51 | 3 | 0 | 3 | non-plum-specific |
| C53 | Cluster_62 | 3 | 0 | 3 | non-plum-specific |
| C54 | Cluster_73 | 3 | 2 | 1 | shared |
| C55 | Cluster_76 | 3 | 0 | 3 | non-plum-specific |
| C56 | Cluster_77 | 3 | 0 | 3 | non-plum-specific |
| C57 | Cluster_78 | 3 | 0 | 3 | non-plum-specific |
| C58 | Cluster_95 | 3 | 3 | 0 | plum-specific |
| C59 | Cluster_10 | 2 | 0 | 2 | non-plum-specific |
| C60 | Cluster_102 | 2 | 0 | 2 | non-plum-specific |
| C61 | Cluster_12 | 2 | 0 | 2 | non-plum-specific |
| C62 | Cluster_13 | 2 | 0 | 2 | non-plum-specific |
| C63 | Cluster_14 | 2 | 0 | 2 | non-plum-specific |
| C64 | Cluster_15 | 2 | 0 | 2 | non-plum-specific |
| C65 | Cluster_16 | 2 | 0 | 2 | non-plum-specific |
| C66 | Cluster_20 | 2 | 1 | 0 | plum-specific |
| C67 | Cluster_23 | 2 | 2 | 0 | plum-specific |
| C68 | Cluster_25 | 2 | 1 | 1 | shared |
| C69 | Cluster_34 | 2 | 0 | 2 | non-plum-specific |
| C70 | Cluster_43 | 2 | 0 | 2 | non-plum-specific |
| C71 | Cluster_52 | 2 | 0 | 2 | non-plum-specific |
| C72 | Cluster_55 | 2 | 0 | 2 | non-plum-specific |
| C73 | Cluster_56 | 2 | 0 | 2 | non-plum-specific |
| C74 | Cluster_57 | 2 | 1 | 1 | shared |
| C75 | Cluster_65 | 2 | 0 | 2 | non-plum-specific |
| C76 | Cluster_83 | 2 | 0 | 2 | non-plum-specific |
| C77 | Cluster_92 | 2 | 2 | 0 | plum-specific |
| C78 | Cluster_1 | 1 | 0 | 1 | non-plum-specific |
| C79 | Cluster_103 | 1 | 0 | 1 | non-plum-specific |
| C80 | Cluster_106 | 1 | 0 | 1 | non-plum-specific |
| C81 | Cluster_109 | 1 | 0 | 1 | non-plum-specific |
| C82 | Cluster_11 | 1 | 0 | 1 | non-plum-specific |
| C83 | Cluster_110 | 1 | 0 | 1 | non-plum-specific |
| C84 | Cluster_111 | 1 | 0 | 1 | non-plum-specific |
| C85 | Cluster_112 | 1 | 0 | 1 | non-plum-specific |
| C86 | Cluster_115 | 1 | 0 | 1 | non-plum-specific |
| C87 | Cluster_116 | 1 | 0 | 1 | non-plum-specific |
| C88 | Cluster_119 | 1 | 0 | 1 | non-plum-specific |
| C89 | Cluster_120 | 1 | 0 | 1 | non-plum-specific |
| C90 | Cluster_123 | 1 | 0 | 1 | non-plum-specific |
| C91 | Cluster_19 | 1 | 1 | 0 | plum-specific |
| C92 | Cluster_2 | 1 | 0 | 1 | non-plum-specific |
| C93 | Cluster_21 | 1 | 1 | 0 | plum-specific |
| C94 | Cluster_22 | 1 | 1 | 0 | plum-specific |
| C95 | Cluster_24 | 1 | 0 | 1 | non-plum-specific |
| C96 | Cluster_3 | 1 | 0 | 1 | non-plum-specific |
| C97 | Cluster_33 | 1 | 0 | 1 | non-plum-specific |
| C98 | Cluster_38 | 1 | 1 | 1 | shared |
| C99 | Cluster_4 | 1 | 0 | 1 | non-plum-specific |
| C100 | Cluster_42 | 1 | 0 | 1 | non-plum-specific |
| C101 | Cluster_44 | 1 | 0 | 1 | non-plum-specific |
| C102 | Cluster_47 | 1 | 0 | 1 | non-plum-specific |
| C103 | Cluster_5 | 1 | 0 | 1 | non-plum-specific |
| C104 | Cluster_53 | 1 | 0 | 1 | non-plum-specific |
| C105 | Cluster_54 | 1 | 0 | 1 | non-plum-specific |
| C106 | Cluster_58 | 1 | 0 | 1 | non-plum-specific |
| C107 | Cluster_63 | 1 | 0 | 1 | non-plum-specific |
| C108 | Cluster_67 | 1 | 0 | 1 | non-plum-specific |
| C109 | Cluster_68 | 1 | 0 | 1 | non-plum-specific |
| C110 | Cluster_69 | 1 | 0 | 1 | non-plum-specific |
| C111 | Cluster_7 | 1 | 0 | 1 | non-plum-specific |
| C112 | Cluster_70 | 1 | 0 | 1 | non-plum-specific |
| C113 | Cluster_71 | 1 | 0 | 1 | non-plum-specific |
| C114 | Cluster_72 | 1 | 0 | 1 | non-plum-specific |
| C115 | Cluster_75 | 1 | 0 | 1 | non-plum-specific |
| C116 | Cluster_79 | 1 | 0 | 1 | non-plum-specific |
| C117 | Cluster_8 | 1 | 0 | 1 | non-plum-specific |
| C118 | Cluster_80 | 1 | 0 | 1 | non-plum-specific |
| C119 | Cluster_81 | 1 | 0 | 1 | non-plum-specific |
| C120 | Cluster_82 | 1 | 0 | 1 | non-plum-specific |
| C121 | Cluster_84 | 1 | 0 | 1 | non-plum-specific |
| C122 | Cluster_89 | 1 | 0 | 1 | non-plum-specific |
| C123 | Cluster_9 | 1 | 0 | 1 | non-plum-specific |
| C124 | Cluster_97 | 1 | 1 | 0 | plum-specific |

**Table S9. Distribution and variability of CAZyme families across *Xylella fastidiosa* subsp. *multiplex* genomes.** Detected carbohydrate-active enzyme (CAZyme) families classified by distribution patterns (conserved, variable presence, broadly absent, or variable copy number). Presence rate indicates the proportion of genomes encoding each family, and copy variation reflects variability in gene copy number. CAZyme families are grouped by functional class.

| CAZyme family | Class | Presence pattern | Presence rate | Copy variation |
| --- | --- | --- | --- | --- |
| AA1 | AA | Conserved | 1 | 0 |
| AA1 | AA | Conserved | 1 | 0 |
| AA4 | AA | Conserved | 1 | 0 |
| AA6 | AA | Conserved | 1 | 0 |
| AA7 | AA | Conserved | 1 | 0.14 |
| CBM2 | CBM | Conserved | 1 | 0.42 |
| CBM32 | CBM | Conserved | 0.98 | 0 |
| CBM63 | CBM | Conserved | 1 | 0 |
| CE11 | CE | Conserved | 1 | 0 |
| CE16 | CE | Conserved | 1 | 0 |
| CE4 | CE | Conserved | 1 | 0 |
| CE9 | CE | Conserved | 1 | 0 |
| GH103 | GH | Conserved | 1 | 0 |
| GH125 | GH | Conserved | 1 | 0 |
| GH144 | GH | Conserved | 1 | 0 |
| GH18 | GH | Conserved | 1 | 0 |
| GH186 | GH | Conserved | 1 | 0 |
| GH2 | GH | Broadly absent | 0.18 | 0 |
| GH20 | GH | Conserved | 1 | 0 |
| GH23 | GH | Conserved | 1 | 0.35 |
| GH24 | GH | Variable copy number | 1 | 2.87 |
| GH25 | GH | Variable presence | 0.71 | 0 |
| GH28 | GH | Conserved | 1 | 0 |
| GH29 | GH | Conserved | 1 | 0 |
| GH3 | GH | Conserved | 1 | 0 |
| GH35 | GH | Conserved | 1 | 0 |
| GH5 | GH | Conserved | 1 | 0.14 |
| GH5 | GH | Conserved | 1 | 0.14 |
| GH6 | GH | Variable presence | 0.8 | 0 |
| GH92 | GH | Conserved | 1 | 0 |
| GT119 | GT | Conserved | 1 | 0 |
| GT121 | GT | Conserved | 1 | 0 |
| GT19 | GT | Conserved | 1 | 0 |
| GT2 | GT | Conserved | 1 | 0.31 |
| GT26 | GT | Conserved | 1 | 0 |
| GT28 | GT | Conserved | 1 | 0 |
| GT30 | GT | Conserved | 1 | 0 |
| GT4 | GT | Conserved | 1 | 0.14 |
| GT5 | GT | Conserved | 0.98 | 0 |
| GT51 | GT | Conserved | 1 | 0.14 |
| GT70 | GT | Conserved | 1 | 0 |
| GT83 | GT | Conserved | 1 | 0 |
| GT9 | GT | Conserved | 1 | 0 |
|  |  |  |  |  |

**Table S10. Within-isolate sequence variation among GH24 protein copies in plum-associated *Xylella fastidiosa* subsp. *multiplex* isolates.** GH24 copies were compared at the predicted protein-sequence level. “Unique protein variants” indicates the number of distinct amino acid sequences identified within each isolate. “Identical copies present” indicates whether two or more GH24 copies within an isolate had identical predicted protein sequences. Across all 15 isolates, 105 GH24 copies represented 35 unique protein sequences; 13 identical protein variants were shared between two or more isolates.

| **Isolate** | **GH24 copies** | **Unique protein variants** | **Identical copies present** |
| --- | --- | --- | --- |
| CFBP13679 | 4 | 4 | No |
| CFBP8070 | 12 | 7 | Yes |
| CFBP8173 | 6 | 3 | Yes |
| CFBP8431 | 13 | 9 | Yes |
| OPR27 | 5 | 5 | No |
| OPR29 | 5 | 5 | No |
| RAAR14 | 1 | 1 | No |
| Riv5 | 5 | 5 | No |
| Tx-2 | 8 | 5 | Yes |
| Tx-467 | 9 | 7 | Yes |
| Tx-468 | 7 | 4 | Yes |
| Tx-470 | 9 | 6 | Yes |
| Tx-471 | 10 | 7 | Yes |
| Tx-9 | 6 | 5 | Yes |
| Tx-B | 5 | 4 | Yes |

**Table S11. Sequence type distribution within prophage clusters across *Xylella fastidiosa* subsp. *multiplex*.** Prophage clusters (C1–C124) annotated with cluster size, host distribution, and associated sequence types (STs). For each cluster, the number of plum and non-plum isolates, ST composition, dominant ST, and distribution pattern (broad, limited, or restricted) are reported, providing insight into lineage-associated patterns of prophage distribution across the *Xfm* dataset.

| **Cluster ID** | **Original cluster** | **Cluster size** | **# plum** | **# nonplum** | **Category** | **# STs** | **STs represented** | **Plum STs** | **Nonplum STs** | **ST distribution** | **Dominant ST** |
| --- | --- | --- | --- | --- | --- | --- | --- | --- | --- | --- | --- |
| C1 | 113 | 39 | 10 | 29 | shared | 9 | ST10; ST26; ST34; ST41; ST6; ST7; ST81; ST88; ST9 | ST10; ST26; ST34; ST41; ST6 | ST41; ST6; ST7; ST81; ST88; ST9 | broad | ST7 |
| C2 | 46 | 36 | 6 | 31 | shared | 8 | ST34; ST42; ST43; ST6; ST7; ST81; ST88; ST9 | ST34; ST6 | ST42; ST43; ST6; ST7; ST81; ST88; ST9 | broad | ST7 |
| C3 | 87 | 34 | 8 | 26 | shared | 11 | ST10; ST26; ST27; ST34; ST42; ST43; ST6; ST7; ST81; ST88; ST9 | ST10; ST26; ST34 | ST27; ST42; ST43; ST6; ST7; ST81; ST88; ST9 | broad | ST7 |
| C4 | 94 | 28 | 4 | 24 | shared | 10 | ST10; ST26; ST27; ST41; ST42; ST43; ST6; ST7; ST81; ST88 | ST10; ST26; ST41; ST6 | ST27; ST41; ST42; ST43; ST6; ST7; ST81; ST88 | broad | ST6 |
| C5 | 121 | 14 | 0 | 12 | non-plum-specific | 4 | ST6; ST7; ST81; ST88 | ST6 | ST6; ST7; ST81; ST88 | broad | ST6 |
| C6 | 91 | 13 | 8 | 1 | shared | 3 | ST26; ST34; ST6 | ST26; ST34 | ST6 | limited | ST26 |
| C7 | 93 | 11 | 5 | 0 | plum-specific | 1 | ST26 | ST26 | - | restricted | ST26 |
| C8 | 30 | 10 | 1 | 10 | shared | 3 | ST6; ST7; ST81 | - | ST6; ST7; ST81 | limited | ST6 |
| C9 | 117 | 9 | 0 | 6 | non-plum-specific | 2 | ST7; ST81 | - | ST7; ST81 | limited | ST81 |
| C10 | 100 | 7 | 4 | 3 | shared | 5 | ST10; ST26; ST6; ST7; ST81 | ST10; ST26; ST6 | ST7; ST81 | broad | ST26 |
| C11 | 104 | 7 | 0 | 6 | non-plum-specific | 3 | ST43; ST7; ST81 | - | ST43; ST7; ST81 | limited | ST7 |
| C12 | 108 | 7 | 0 | 7 | non-plum-specific | 2 | ST7; ST81 | - | ST7; ST81 | limited | ST81 |
| C13 | 114 | 7 | 0 | 7 | non-plum-specific | 2 | ST7; ST81 | - | ST7; ST81 | limited | ST81 |
| C14 | 118 | 7 | 0 | 7 | non-plum-specific | 2 | ST7; ST81 | - | ST7; ST81 | limited | ST81 |
| C15 | 29 | 7 | 0 | 5 | non-plum-specific | 2 | ST6; ST7 | ST6 | ST6; ST7 | limited | ST6 |
| C16 | 40 | 7 | 3 | 5 | shared | 3 | ST34; ST6; ST7 | ST34 | ST6; ST7 | limited | ST6 |
| C17 | 86 | 7 | 5 | 1 | shared | 2 | ST34; ST9 | ST34 | ST9 | limited | ST34 |
| C18 | 101 | 6 | 6 | 0 | plum-specific | 2 | ST10; ST26 | ST10; ST26 | - | limited | ST26 |
| C19 | 122 | 6 | 0 | 5 | non-plum-specific | 2 | ST7; ST81 | - | ST7; ST81 | limited | ST81 |
| C20 | 28 | 6 | 1 | 6 | shared | 2 | ST6; ST7 | ST6 | ST6; ST7 | limited | ST6 |
| C21 | 32 | 6 | 0 | 6 | non-plum-specific | 3 | ST6; ST7; ST88 | ST6 | ST6; ST7; ST88 | limited | ST6 |
| C22 | 37 | 6 | 1 | 6 | shared | 2 | ST6; ST7 | ST6 | ST6; ST7 | limited | ST6 |
| C23 | 39 | 6 | 1 | 5 | shared | 2 | ST6; ST7 | - | ST6; ST7 | limited | ST6 |
| C24 | 6 | 6 | 4 | 2 | shared | 4 | ST10; ST26; ST27; ST43 | ST10; ST26 | ST27; ST43 | broad | ST26 |
| C25 | 60 | 6 | 1 | 6 | shared | 2 | ST6; ST7 | ST6 | ST6; ST7 | limited | ST6 |
| C26 | 61 | 6 | 0 | 3 | non-plum-specific | 1 | ST7 | - | ST7 | restricted | ST7 |
| C27 | 66 | 6 | 1 | 5 | shared | 3 | ST26; ST6; ST7 | ST26 | ST6; ST7 | limited | ST7 |
| C28 | 96 | 6 | 4 | 0 | plum-specific | 1 | ST26 | ST26 | - | restricted | ST26 |
| C29 | 105 | 5 | 3 | 2 | shared | 3 | ST26; ST7; ST81 | ST26 | ST7; ST81 | limited | ST26 |
| C30 | 48 | 5 | 0 | 1 | non-plum-specific | 1 | ST6 | - | ST6 | restricted | ST6 |
| C31 | 85 | 5 | 5 | 0 | plum-specific | 1 | ST34 | ST34 | - | restricted | ST34 |
| C32 | 88 | 5 | 0 | 4 | non-plum-specific | 2 | ST6; ST81 | - | ST6; ST81 | limited | ST6 |
| C33 | 98 | 5 | 5 | 0 | plum-specific | 1 | ST26 | ST26 | - | restricted | ST26 |
| C34 | 124 | 4 | 0 | 4 | non-plum-specific | 2 | ST7; ST81 | - | ST7; ST81 | limited | ST7 |
| C35 | 17 | 4 | 3 | 1 | shared | 3 | ST10; ST26; ST6 | ST10; ST26 | ST6 | limited | ST10 |
| C36 | 26 | 4 | 2 | 2 | shared | 2 | ST26; ST41 | ST26; ST41 | ST41 | limited | ST26 |
| C37 | 27 | 4 | 0 | 4 | non-plum-specific | 3 | ST27; ST41; ST42 | ST41 | ST27; ST41; ST42 | limited | ST41 |
| C38 | 31 | 4 | 0 | 4 | non-plum-specific | 2 | ST6; ST7 | - | ST6; ST7 | limited | ST6 |
| C39 | 36 | 4 | 0 | 4 | non-plum-specific | 2 | ST6; ST7 | - | ST6; ST7 | limited | ST7 |
| C40 | 41 | 4 | 0 | 3 | non-plum-specific | 2 | ST6; ST81 | - | ST6; ST81 | limited | ST6 |
| C41 | 45 | 4 | 3 | 1 | shared | 2 | ST34; ST81 | ST34 | ST81 | limited | ST34 |
| C42 | 59 | 4 | 1 | 4 | shared | 3 | ST6; ST7; ST88 | ST6 | ST6; ST7; ST88 | limited | ST6 |
| C43 | 64 | 4 | 0 | 4 | non-plum-specific | 2 | ST6; ST7 | - | ST6; ST7 | limited | ST6 |
| C44 | 74 | 4 | 3 | 1 | shared | 2 | ST34; ST9 | ST34 | ST9 | limited | ST34 |
| C45 | 90 | 4 | 3 | 0 | plum-specific | 2 | ST10; ST26 | ST10; ST26 | - | limited | ST26 |
| C46 | 99 | 4 | 3 | 0 | plum-specific | 1 | ST26 | ST26 | - | restricted | ST26 |
| C47 | 107 | 3 | 1 | 2 | shared | 3 | ST10; ST81; ST9 | ST10 | ST81; ST9 | limited | ST10 |
| C48 | 18 | 3 | 3 | 0 | plum-specific | 2 | ST10; ST26 | ST10; ST26 | - | limited | ST26 |
| C49 | 35 | 3 | 0 | 3 | non-plum-specific | 1 | ST7 | - | ST7 | restricted | ST7 |
| C50 | 49 | 3 | 0 | 3 | non-plum-specific | 2 | ST6; ST81 | - | ST6; ST81 | limited | ST6 |
| C51 | 50 | 3 | 0 | 3 | non-plum-specific | 2 | ST6; ST81 | - | ST6; ST81 | limited | ST6 |
| C52 | 51 | 3 | 0 | 3 | non-plum-specific | 2 | ST6; ST81 | - | ST6; ST81 | limited | ST6 |
| C53 | 62 | 3 | 0 | 3 | non-plum-specific | 1 | ST7 | - | ST7 | restricted | ST7 |
| C54 | 73 | 3 | 2 | 1 | shared | 2 | ST26; ST9 | ST26 | ST9 | limited | ST26 |
| C55 | 76 | 3 | 0 | 3 | non-plum-specific | 2 | ST41; ST9 | ST41 | ST41; ST9 | limited | ST41 |
| C56 | 77 | 3 | 0 | 3 | non-plum-specific | 1 | ST7 | - | ST7 | restricted | ST7 |
| C57 | 78 | 3 | 0 | 3 | non-plum-specific | 1 | ST7 | - | ST7 | restricted | ST7 |
| C58 | 95 | 3 | 3 | 0 | plum-specific | 1 | ST26 | ST26 | - | restricted | ST26 |
| C59 | 10 | 2 | 0 | 2 | non-plum-specific | 2 | ST27; ST43 | - | ST27; ST43 | limited | ST27 |
| C60 | 102 | 2 | 0 | 2 | non-plum-specific | 2 | ST7; ST81 | - | ST7; ST81 | limited | ST7 |
| C61 | 12 | 2 | 0 | 2 | non-plum-specific | 1 | ST41 | ST41 | ST41 | restricted | ST41 |
| C62 | 13 | 2 | 0 | 2 | non-plum-specific | 1 | ST41 | ST41 | ST41 | restricted | ST41 |
| C63 | 14 | 2 | 0 | 2 | non-plum-specific | 1 | ST41 | ST41 | ST41 | restricted | ST41 |
| C64 | 15 | 2 | 0 | 2 | non-plum-specific | 1 | ST41 | ST41 | ST41 | restricted | ST41 |
| C65 | 16 | 2 | 0 | 2 | non-plum-specific | 1 | ST41 | ST41 | ST41 | restricted | ST41 |
| C66 | 20 | 2 | 1 | 0 | plum-specific | 1 | ST10 | ST10 | - | restricted | ST10 |
| C67 | 23 | 2 | 2 | 0 | plum-specific | 2 | ST10; ST26 | ST10; ST26 | - | limited | ST10 |
| C68 | 25 | 2 | 1 | 1 | shared | 2 | ST26; ST27 | ST26 | ST27 | limited | ST26 |
| C69 | 34 | 2 | 0 | 2 | non-plum-specific | 1 | ST7 | - | ST7 | restricted | ST7 |
| C70 | 43 | 2 | 0 | 2 | non-plum-specific | 2 | ST6; ST81 | - | ST6; ST81 | limited | ST6 |
| C71 | 52 | 2 | 0 | 2 | non-plum-specific | 2 | ST43; ST6 | - | ST43; ST6 | limited | ST43 |
| C72 | 55 | 2 | 0 | 2 | non-plum-specific | 1 | ST43 | - | ST43 | restricted | ST43 |
| C73 | 56 | 2 | 0 | 2 | non-plum-specific | 1 | ST7 | - | ST7 | restricted | ST7 |
| C74 | 57 | 2 | 1 | 1 | shared | 2 | ST26; ST7 | ST26 | ST7 | limited | ST26 |
| C75 | 65 | 2 | 0 | 2 | non-plum-specific | 2 | ST6; ST7 | - | ST6; ST7 | limited | ST6 |
| C76 | 83 | 2 | 0 | 2 | non-plum-specific | 1 | ST7 | - | ST7 | restricted | ST7 |
| C77 | 92 | 2 | 2 | 0 | plum-specific | 1 | ST26 | ST26 | - | restricted | ST26 |
| C78 | 1 | 1 | 0 | 1 | non-plum-specific | 1 | ST42 | - | ST42 | restricted | ST42 |
| C79 | 103 | 1 | 0 | 1 | non-plum-specific | 1 | ST81 | - | ST81 | restricted | ST81 |
| C80 | 106 | 1 | 0 | 1 | non-plum-specific | 1 | ST81 | - | ST81 | restricted | ST81 |
| C81 | 109 | 1 | 0 | 1 | non-plum-specific | 1 | ST7 | - | ST7 | restricted | ST7 |
| C82 | 11 | 1 | 0 | 1 | non-plum-specific | 1 | ST43 | - | ST43 | restricted | ST43 |
| C83 | 110 | 1 | 0 | 1 | non-plum-specific | 1 | ST7 | - | ST7 | restricted | ST7 |
| C84 | 111 | 1 | 0 | 1 | non-plum-specific | 1 | ST7 | - | ST7 | restricted | ST7 |
| C85 | 112 | 1 | 0 | 1 | non-plum-specific | 1 | ST7 | - | ST7 | restricted | ST7 |
| C86 | 115 | 1 | 0 | 1 | non-plum-specific | 1 | ST81 | - | ST81 | restricted | ST81 |
| C87 | 116 | 1 | 0 | 1 | non-plum-specific | 1 | ST81 | - | ST81 | restricted | ST81 |
| C88 | 119 | 1 | 0 | 1 | non-plum-specific | 1 | ST81 | - | ST81 | restricted | ST81 |
| C89 | 120 | 1 | 0 | 1 | non-plum-specific | 1 | ST81 | - | ST81 | restricted | ST81 |
| C90 | 123 | 1 | 0 | 1 | non-plum-specific | 1 | ST81 | - | ST81 | restricted | ST81 |
| C91 | 19 | 1 | 1 | 0 | plum-specific | 1 | ST10 | ST10 | - | restricted | ST10 |
| C92 | 2 | 1 | 0 | 1 | non-plum-specific | 1 | ST27 | - | ST27 | restricted | ST27 |
| C93 | 21 | 1 | 1 | 0 | plum-specific | 1 | ST10 | ST10 | - | restricted | ST10 |
| C94 | 22 | 1 | 1 | 0 | plum-specific | 1 | ST10 | ST10 | - | restricted | ST10 |
| C95 | 24 | 1 | 0 | 1 | non-plum-specific | 1 | ST27 | - | ST27 | restricted | ST27 |
| C96 | 3 | 1 | 0 | 1 | non-plum-specific | 1 | ST27 | - | ST27 | restricted | ST27 |
| C97 | 33 | 1 | 0 | 1 | non-plum-specific | 1 | ST6 | ST6 | - | restricted | ST6 |
| C98 | 38 | 1 | 1 | 1 | shared | 1 | ST6 | - | ST6 | restricted | ST6 |
| C99 | 4 | 1 | 0 | 1 | non-plum-specific | 1 | ST27 | - | ST27 | restricted | ST27 |
| C100 | 42 | 1 | 0 | 1 | non-plum-specific | 1 | ST6 | - | ST6 | restricted | ST6 |
| C101 | 44 | 1 | 0 | 1 | non-plum-specific | 1 | ST81 | - | ST81 | restricted | ST81 |
| C102 | 47 | 1 | 0 | 1 | non-plum-specific | 1 | ST81 | - | ST81 | restricted | ST81 |
| C103 | 5 | 1 | 0 | 1 | non-plum-specific | 1 | ST27 | - | ST27 | restricted | ST27 |
| C104 | 53 | 1 | 0 | 1 | non-plum-specific | 1 | ST43 | - | ST43 | restricted | ST43 |
| C105 | 54 | 1 | 0 | 1 | non-plum-specific | 1 | ST43 | - | ST43 | restricted | ST43 |
| C106 | 58 | 1 | 0 | 1 | non-plum-specific | 1 | ST7 | - | ST7 | restricted | ST7 |
| C107 | 63 | 1 | 0 | 1 | non-plum-specific | 1 | ST6 | - | ST6 | restricted | ST6 |
| C108 | 67 | 1 | 0 | 1 | non-plum-specific | 1 | ST88 | - | ST88 | restricted | ST88 |
| C109 | 68 | 1 | 0 | 1 | non-plum-specific | 1 | ST88 | - | ST88 | restricted | ST88 |
| C110 | 69 | 1 | 0 | 1 | non-plum-specific | 1 | ST88 | - | ST88 | restricted | ST88 |
| C111 | 7 | 1 | 0 | 1 | non-plum-specific | 1 | ST43 | - | ST43 | restricted | ST43 |
| C112 | 70 | 1 | 0 | 1 | non-plum-specific | 1 | ST88 | - | ST88 | restricted | ST88 |
| C113 | 71 | 1 | 0 | 1 | non-plum-specific | 1 | ST9 | - | ST9 | restricted | ST9 |
| C114 | 72 | 1 | 0 | 1 | non-plum-specific | 1 | ST9 | - | ST9 | restricted | ST9 |
| C115 | 75 | 1 | 0 | 1 | non-plum-specific | 1 | ST9 | - | ST9 | restricted | ST9 |
| C116 | 79 | 1 | 0 | 1 | non-plum-specific | 1 | ST7 | - | ST7 | restricted | ST7 |
| C117 | 8 | 1 | 0 | 1 | non-plum-specific | 1 | ST43 | - | ST43 | restricted | ST43 |
| C118 | 80 | 1 | 0 | 1 | non-plum-specific | 1 | ST7 | - | ST7 | restricted | ST7 |
| C119 | 81 | 1 | 0 | 1 | non-plum-specific | 1 | ST7 | - | ST7 | restricted | ST7 |
| C120 | 82 | 1 | 0 | 1 | non-plum-specific | 1 | ST7 | - | ST7 | restricted | ST7 |
| C121 | 84 | 1 | 0 | 1 | non-plum-specific | 1 | ST7 | - | ST7 | restricted | ST7 |
| C122 | 89 | 1 | 0 | 1 | non-plum-specific | 1 | ST81 | - | ST81 | restricted | ST81 |
| C123 | 9 | 1 | 0 | 1 | non-plum-specific | 1 | ST43 | - | ST43 | restricted | ST43 |
| C124 | 97 | 1 | 1 | 0 | plum-specific | 1 | ST26 | ST26 | - | restricted | ST26 |
